# Supplementary material for: Rac1 Controls Both the Secretory Function of the Mammary Gland and Its Remodeling for Successive Gestations
Source: Dev Cell. 2016 Sep 12;38(5):522–35. doi: 10.1016/j.devcel.2016.08.005 (PMC5022528; doi:10.1016/j.devcel.2016.08.005)
Supplement: Document S1. Supplemental Experimental Procedures, Figures S1–S7, and Tables S1–S3 [file mmc1.pdf]

Developmental Cell, Volume *38*

Supplemental Information

Rac1 Controls Both the Secretory Function of the Mammary Gland and Its Remodeling for Successive Gestations

Nasreen Akhtar, Weiping Li, Aleksander Mironov, and Charles H. Streuli

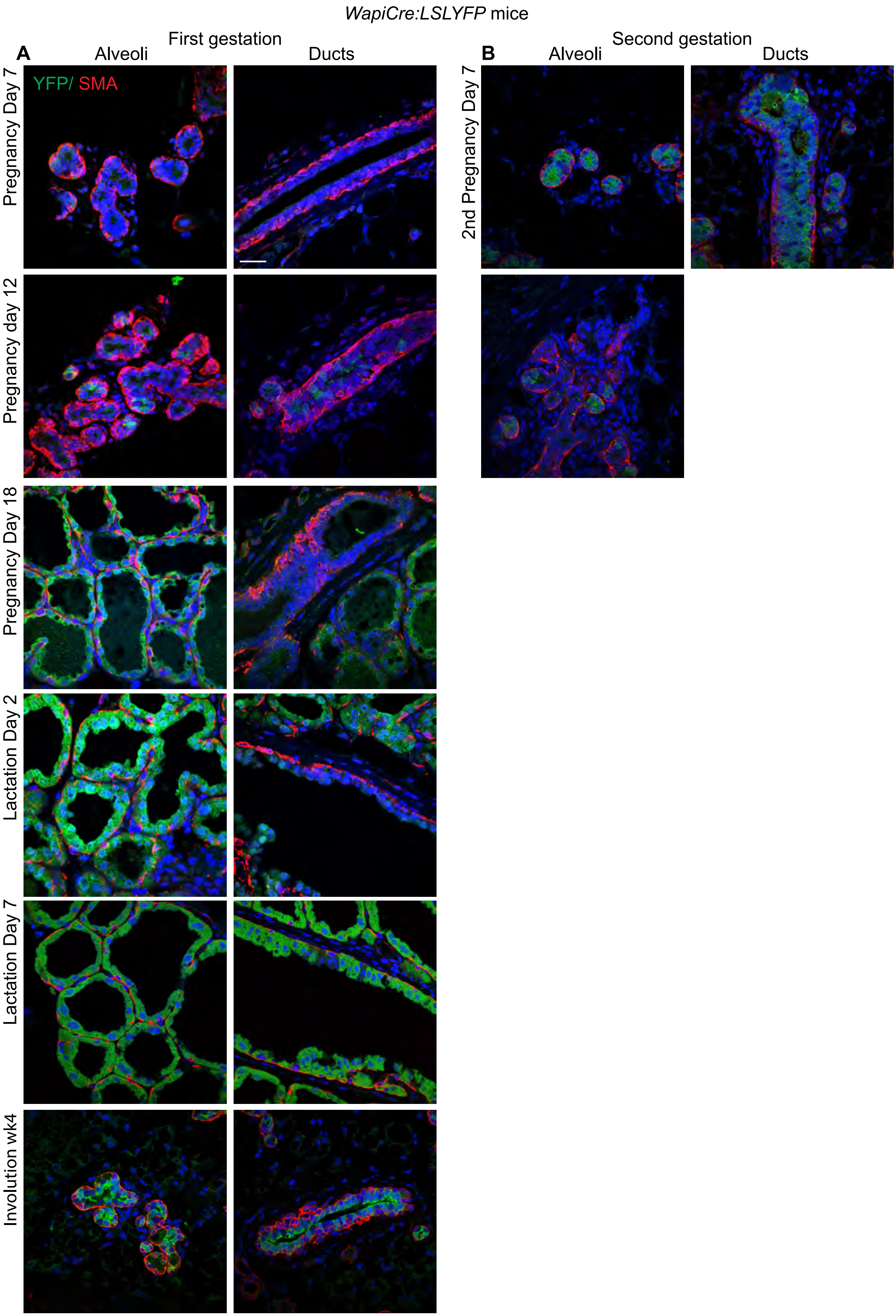
WapiCre:LSLYFP mice
First gestation
A
Alveoli
Ducts
Second gestation
B
Alveoli
Ducts
YFP/ SMA
Pregnancy Day 7
Pregnancy day 12
Pregnancy Day 18
Lactation Day 2
Lactation Day 7
Involution wk4
2nd Pregnancy Day 7

Figure S1

Figure S1 related to Fig 1-3: *WAPiCre mediated recombination during different stages of mammary gland development.*

WAPiCre mediated recombination was detected in the mammary gland at various stages of pregnancy and lactation in the first and second cycle, using a YFP reporter transgenic mouse line (*WAPiCre:LSLYFP*).

(A) GFP immunostaining was used to detect YFP reporter gene expression in alveoli (left panel) and ducts (right panel) in the first cycle at pregnancy (P) days 7, 12, 18, lactation day 2, 7 and 4 weeks post-lactational involution. Smooth muscle actin (SMA) immunostaining was used to detect myoepithelia. Extensive *WAPiCre* mediated recombination was detected within alveoli in late pregnancy (P18) after alveologenesis had proceeded and within ducts around lactation day 7. YFP reporter gene expression was still evident in glands 4 weeks post-lactational involution.

(B) In the second cycle, new alveoli detected at P7 arise from stem/progenitors that have undergone recombination in the first cycle (top left, YFP positive) but also from stem/progenitor populations that escaped recombination in the first cycle (bottom panel, absence of YFP in 20-30% of the gland).

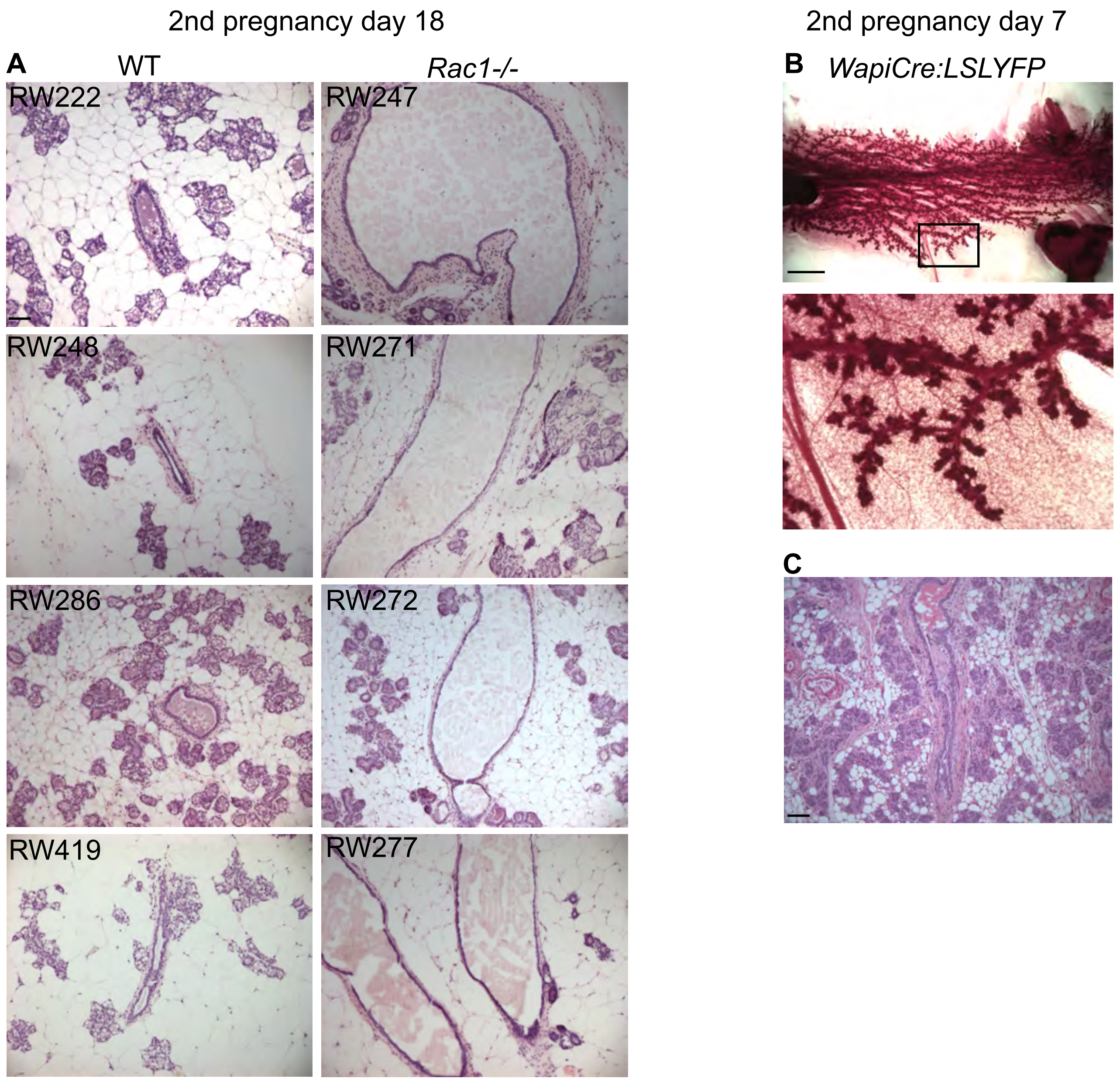

Figure S2 related to Fig 1: *Baobab duct phenotype develops specifically in Rac1-deficient mammary glands.*
(A) Representative Haematoxylin and Eosin stains of n=4 mice showing gross dilation of mammary ducts in day 18 of a second pregnancy. Bar: 100µm. RW (RacWAP) numbers = mouse identification in colony.
(B) Carmine staining of wholemounted *WAPiCre:LSLYFP* glands expressing WT Rac1 alleles show normal alveolar and ductal morphogenesis in a second pregnancy. Day 7 of pregnancy were used to visualise mammary ducts more clearly. Bar: 5mm.
(C) Haematoxylin and Eosin stain of glands as in (B). Bar: 100µm.

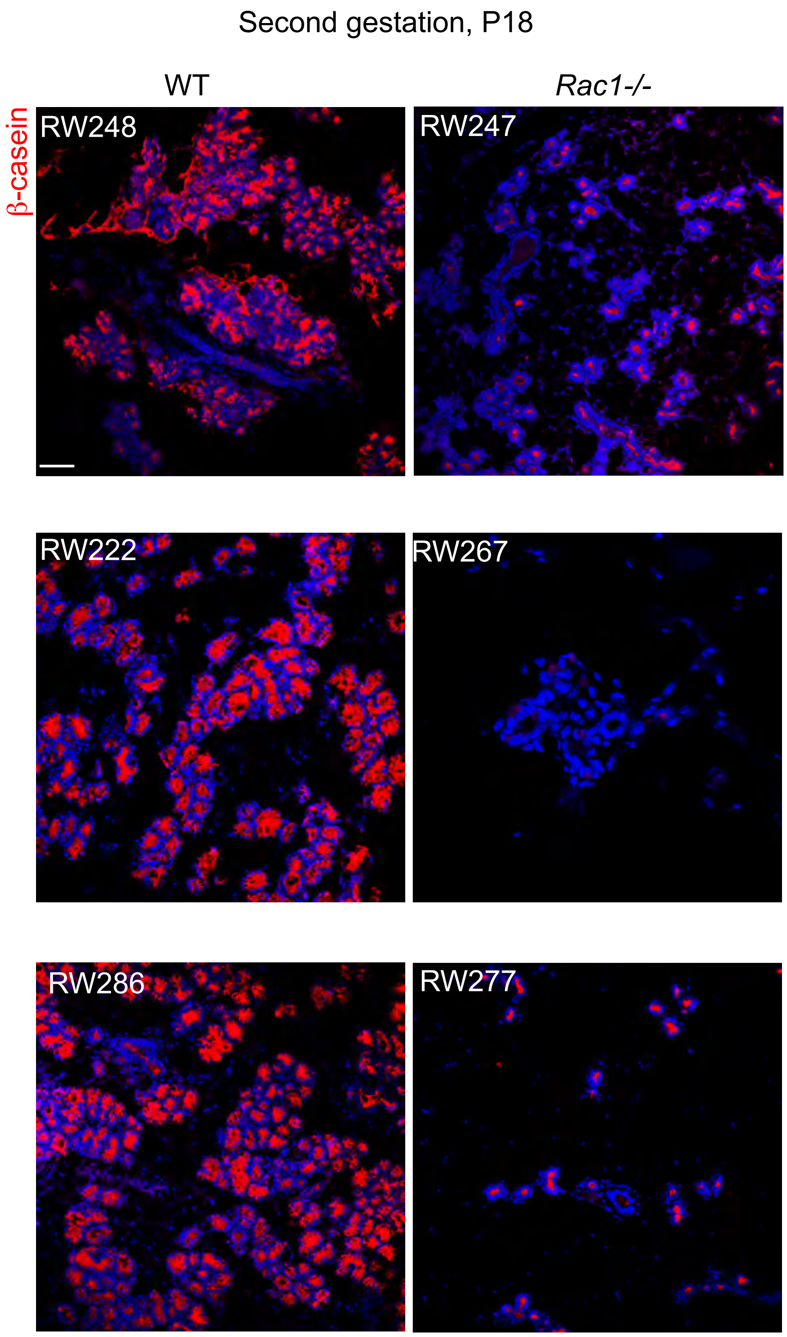

Figure S3 related to Fig 2: *Lactation defect in Rac1-/- mammary glands.*
Representative β-casein immunofluorescence stains of n=3 mice showing defective lactation at day 18 of a second pregnancy. Earclip numbers are indicated on micrographs. Bar: 150μm.

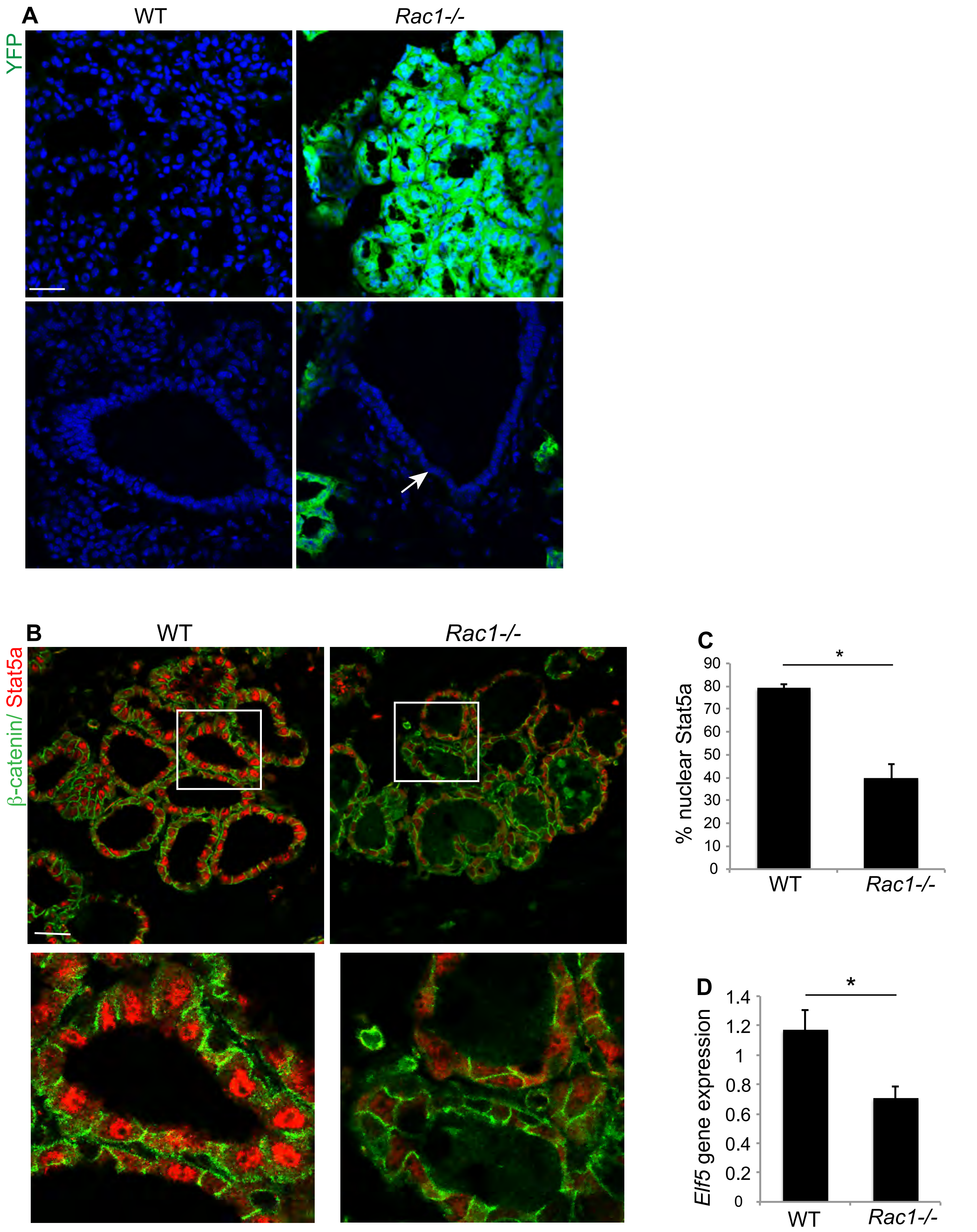
A
WT
Rac1-/-
YFP
B
WT
Rac1-/-
β-catenin/ Stat5a
C
% nuclear Stat5a
90
80
70
60
50
40
30
20
10
0
*
WT
Rac1-/-
D
Elf5 gene expression
1.4
1.2
1
0.8
0.6
0.4
0.2
0
*
WT
Rac1-/-

Figure S4

Figure S4 related to Fig 3: *Rac1 ablation and milk transcription factor activity in the first cycle.*
A) P18 WT and *Rac1-/-* glands, immuno-stained for YFP reporter gene expression. The presence of YFP in *Rac1-/-* glands showed that Cre-mediated recombination occurred in alveoli but not ducts. Arrow points to a genetically WT duct in *transgenic* tissue. Bar: 45mm.
(B) Immunofluorescence staining of Stat5a at lactation day 2 reveals reduced nuclear translocation in *Rac1-/-* alveoli. b-catenin was used to mark cell edges. Bar: 15mm (insert: 7mm).
(C) Quantitative analysis of Stat5a nuclear translocation. Nine areas/mouse were analysed. Error bars; +/- SEM of n=3 mice per group. *P=<0.05.
(D) Quantitative RT-PCR shows reduced Elf5 gene expression in P18 *Rac1-/-* glands. Error bars: +/- SEM of n=3 mice. * P<0.05

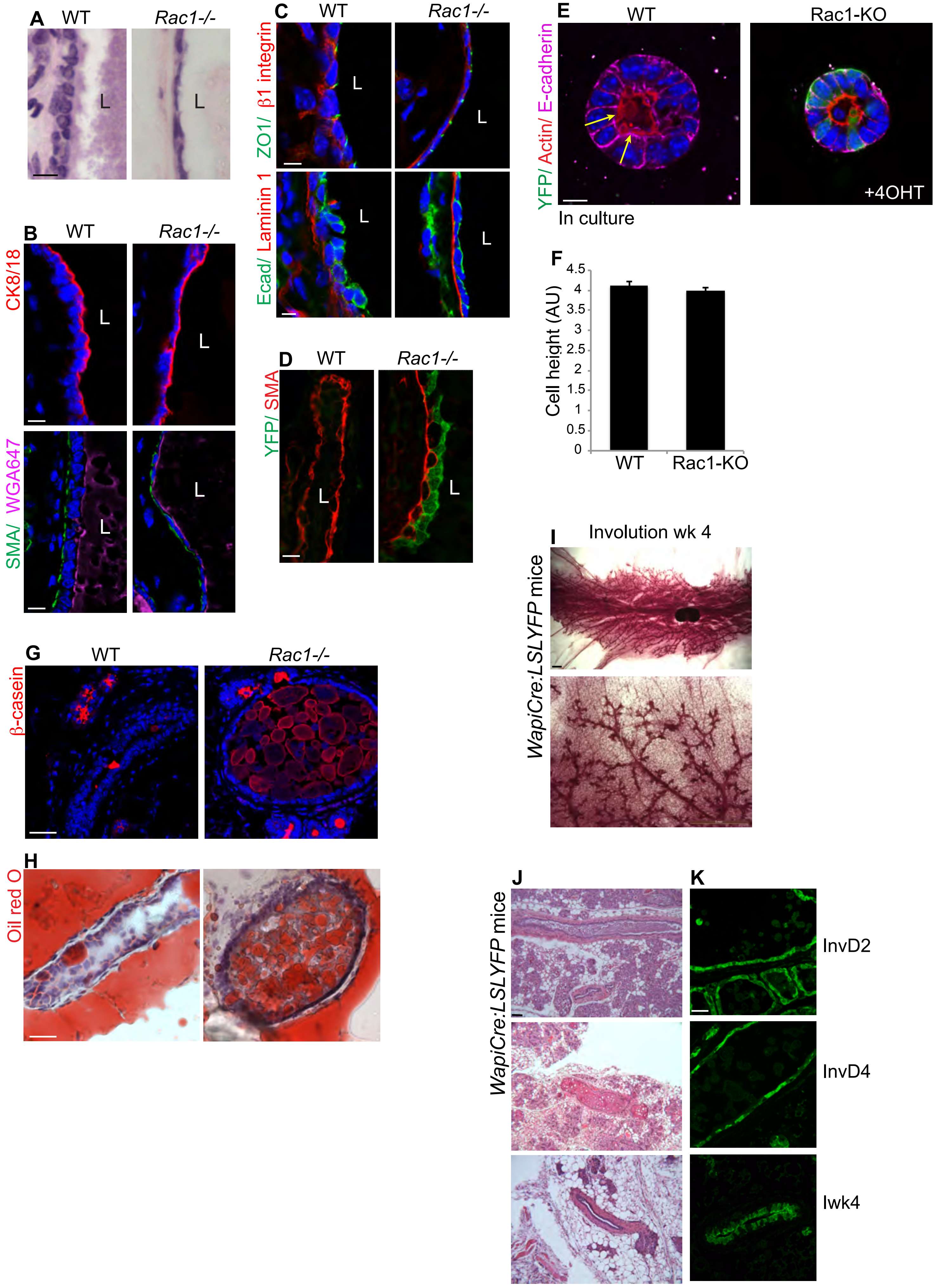
A
WT
Rac1-/-
L
B
CK8/18
SMA/ WGA647
C
ZO1/ β1 integrin
Ecad/ Laminin 1
D
YFP/ SMA
E
YFP/ Actin/ E-cadherin
Rac1-KO
+4OHT
In culture
F
Cell height (AU)
4.5
4
3.5
3
2.5
2
1.5
1
0.5
0
Rac1-KO
G
β-casein
H
Oil red O
I
Involution wk 4
WapiCre:LSLYFP mice
J
K
InvD2
InvD4
Iwk4

Figure S5

Figure S5 related to Fig 4: *Characterisation of epithelial architecture in baobab ducts.*
(A) Haematoxylin and Eosin stain shows that *Rac1-/-* baobab ducts exhibit flattened epithelia.
(B) Baobab ducts are composed of a bi-layer of luminal and myoepithelial cells. Keratin 8/18 and WGA-488 were used to detect luminal cells and smooth muscle actin (SMA) to detect myoepithelia.
(C) Apicobasal polarity is still intact in *Rac1-/-* baobab ducts.
(D) YFP reporter gene expression shows that Rac1 ablation is restricted to luminal epithelia in baobab ducts.
(E) Representative confocal image of WT and Rac1-KO acini used to measure cell height. Actin and E-cadherin was used to demark cell edges. Bar; 10mm.
(F) Quantification of cell height in WT and Rac1-KO acini. Error bars; +/-SEM of 99 cell heights per condition.
(G) Immunofluorescence stain with b-casein showing milk products in baobab duct lumens.
(H) Oil red O stain showing accumulation of milk fat globules in baobab duct lumens.
L= lumen. Bar: (a-d) 5mm, (e) 10mm, (g,h) 40mm.
(I) Carmine staining of whole-mounted mammary glands from mice at 4 weeks post-lactational involution shows no bloating of ducts. Bar: 2.8 mm.
(J) H+E stain of mammary glands at involution day 2,4 and 4 weeks. Bar: 100mm.
(K) Immunofluorescence staining of YFP shows efficient WAPiCre mediated recombination in *WAPiCre:LSLYFP* mice. Bar: 45mm.

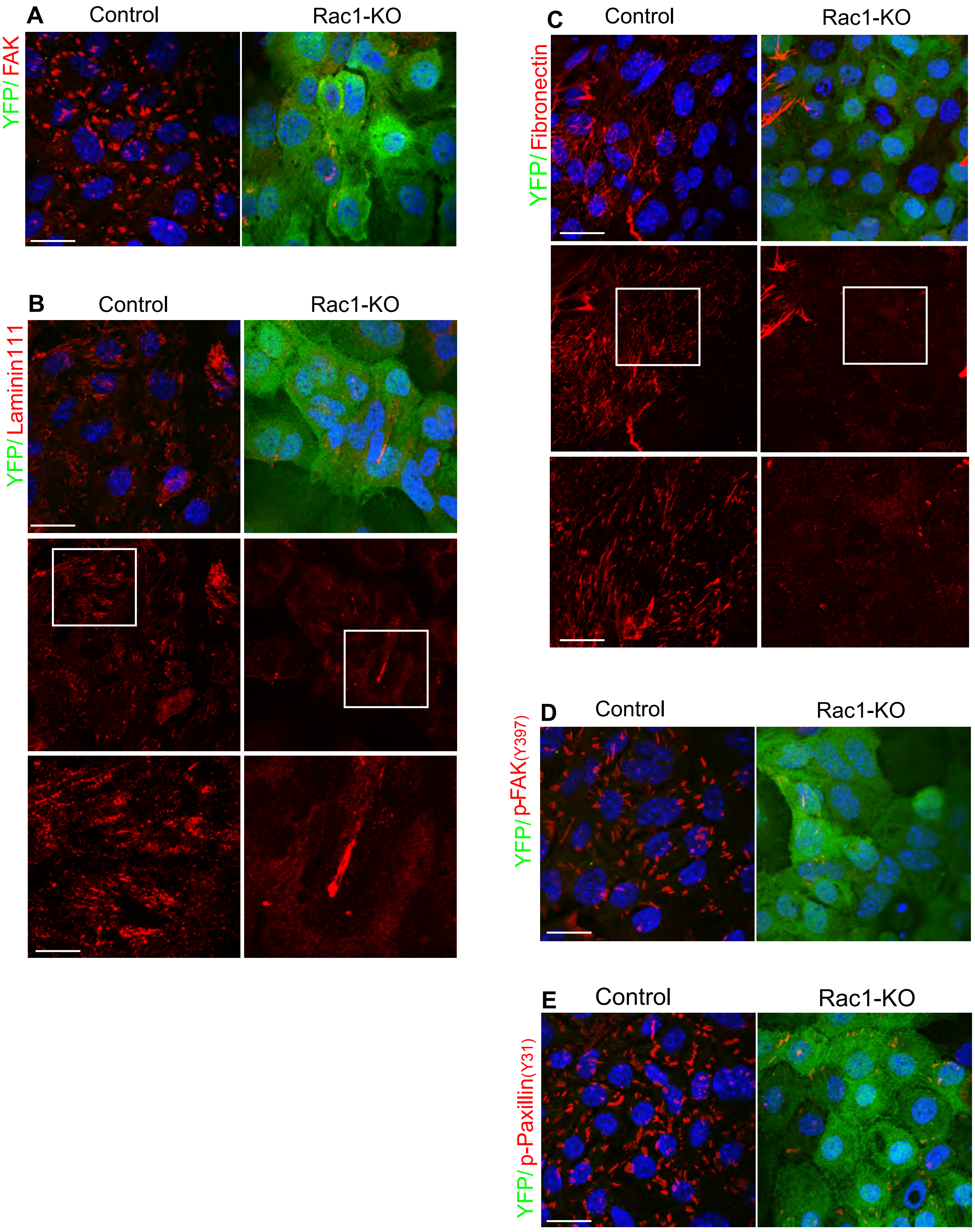

Figure S6 related to Fig 5: *Reduced cell-ECM adhesion in Rac1 depleted MECs.*
(A-E) GFP antibody stain was used to detect YFP expression in Rac1-KO MECs.
(A) Immunostaining with FAK shows markedly reduced focal adhesion complexes in Rac1-KO MECs in culture.
(B) Reduced laminin111 organisation within focal adhesions in Rac1-KO MECs.
(C) Reduced fibronectin organisation within focal adhesions in Rac1-KO MECs.
(D) Immunofluorescence stain with phosphorylated FAK (Y397).
(E) Immunofluorescence stain with phosphorylated paxillin (Y31).
Bar: 10µm, (inset 4µm).

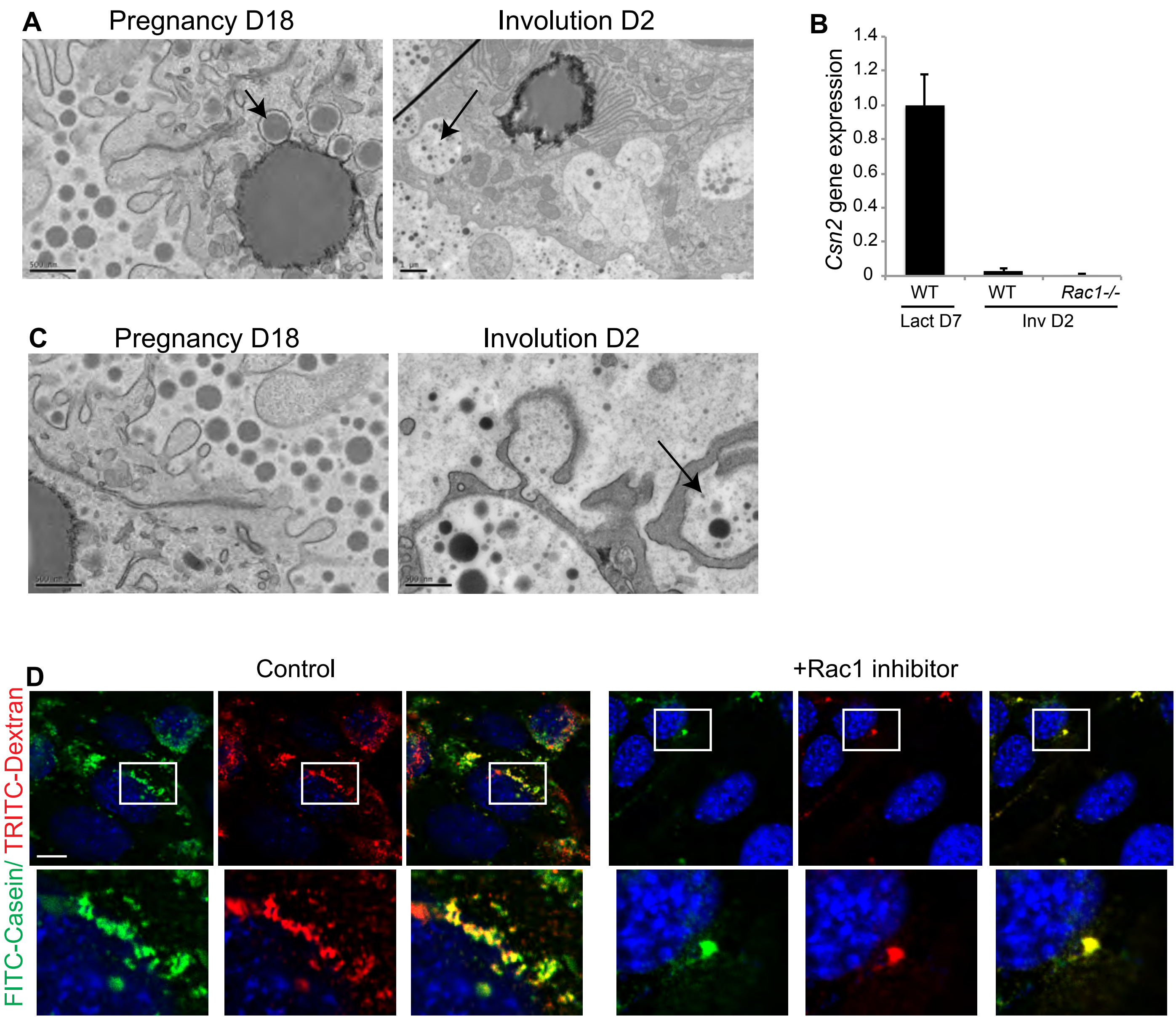

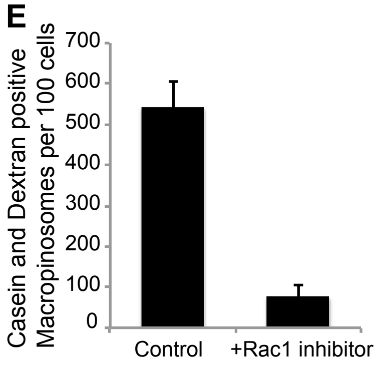

F

Gene	Fold change	P-Value
TYRO3 protein tyrosine kinase 3	-4.03	6.27E-04
CD36 antigen	-3.12	1.42E-02
CD14 antigen	-2.16	1.10E-01
milk fat globule-EGF factor 8 protein	-2.35	1.17E-01
AXL receptor tyrosine kinase	-1.30	2.88E-01

Figure S7

Figure S7 related to Fig 6: *Impaired engulfment in the absence of Rac1.*
(A) Electron micrograph of *left;* pregnancy day 18 and *right;* involution day 2 mammary glands. Arrows points to milk micelles in the cell. Note; milk micelles before secretion are encapsulated with a membrane (left). Bar: *left*; 500nm and *right*; 1mm.
(B) Quantitative RT-PCR for *Csn2* gene expression, showing virtually no new milk synthesis at involution day 2, compared with Lactation day 7. Error bars; +/-SEM of n=3 mice.
(C) Electron micrograph showing milk-engulfing macropinosomes (arrow) at the apical membrane in involution day 2 glands. These were not detected in secretory MECs at pregnancy day 18. Bar: 500nm.
(D) Macropinocytic engulfment of FITC conjugated casein in MEC cultures was drastically impaired upon inhibition of Rac1. Cells were treated with DMSO vehicle only (control), or 50mM NSC23766 Rac1 inhibitor overnight, prior to measurement of macropinocytosis. 70k Mr TRITC–dextran was used as a positive marker of macropinocytic engulfment. Bar: 10mm.
(E) Quantification of (A), casein and dextran positive macropinosomes were counted in 100 cells for each condition. Error bars; +/-SEM of n=3.
(F) Gene expression data from WT and *Rac1-/-* Involution day 2 glands showing down-regulation of engulfment receptor gene sets in *transgenics*, n=3 mice were used per condition.

Gene symbol	Gene description	FC	P. Value
Csn1s2a	casein alpha s2-like A	-38.63 to -45.05	1.90E-05 to 1.43E-03
Wap	whey acidic protein	-10.25	2.55E-05
Csn2	casein beta	-2.12	2.93E-03
Csnk1g3	casein kinase 1, gamma 3	-2.95	1.00E-02
Csn3	casein kappa	-4.05	1.41E-02
Csn1s2b	casein alpha s2-like B	-1.89	2.15E-02
Csnk1a1	casein kinase 1, alpha 1	-2.32	3.02E-02
Csn1s1	casein alpha s1	-4.02	4.93E-02
Btn1a1	butyrophilin, subfamily 1, member A1	-5.21	2.89E-03
Xdh	xanthine dehydrogenase	-3.86	2.03E-03
Lalba	lactalbumin alpha	-5.35	4.52E-03

Table S1 related to Fig 2: *List of lactation genes down-regulated in Rac1-/- glands.*
Microarray gene expression data from WT and *Rac1-/-* mammary glands, showing list of milk components down-regulated in *transgenics*, n=3 mice were used per condition.

Gene symbol	Gene description	FC	P. Value
Elf5	E74-like factor 5	-3.14	5.65E-03
Stat5a	signal transducer and activator of transcription 5A	-3.65	8.54E-03
Stat5b	signal transducer and activator of transcription 5B	-2.30	3.56E-02
Gata 3	GATA binding protein 3	-1.80	1.16E-01
Prlr	prolactin receptor	-1.63	3.53E-02

Table S2 related to Fig 2: *List of lactation signaling pathway genes down-regulated in Rac1-/- glands.* Microarray gene expression data from WT and *Rac1-/-* mammary glands, showing list of lactation signaling pathway components and transcription factors down-regulated in *transgenics*, n=3 mice were used per condition.

Gene	Fold Change in *Rac1-/-*
CCL7	2.61
CCL2	2.83
CCL3	2.04
CCL19	2.12
CCL22	1.44
CXCL16	1.27
CCL27	1.17
CCL4	1.38
CXCL2	1.92
CCL20	1.30
CCL8	1.57
CXCL10	1.33
CXCL13	1.10
CXCL1	1.08
CCL24	1.26
CCL17	1.30
CCL25	1.27
CCL11	1.17
CXCL11	1.16
CXCL1	1.68

Table S3 related to Fig 7: *List of chemokines up-regulated in Rac1-/- glands at involution day 2.*
(A) Microarray gene expression data from WT and *Rac1-/-* Involution day 2 mammary glands, n=3 mice were used per condition.

Supplemental Experimental Procedures

**ANTIBODY**	**SOURCE**	**IF**	**IHC**	**IB**
Adipophilin	Progen #GP40	1;200		
beta1 integrin	Millipore #MAB 1997	1;100		
beta-casein	in house		1;1000	1;5000
beta-casein-RRX conjugate	in house	1;200		
beta-catenin	BD Biosciences #610154	1;200		
Calnexin	Bioquote SPA-#SPC-108A/B			1;4000
Cleaved caspase-3	Cell Signalling Technology #9661	1;100		1;50
Cytokeratin 8/18	Progen #GP11	1;200		
E-cadherin (clone ECCD-2)	Life Technologies	1;100		
E-cadherin (clone 4A2)	Cell Signalling Technology mAb #14472			1;5000
Focal adhesion kinase	BD Biosciences #610088	1;100		
FAK (Y397)	Biosource Int. #44624-G	1;100		
Fibronectin	Millipore #AB2033	1;100		
F4:80 macrophage	Serotec #MCA497GA	1;200		
Green fluorescent protein	Invitrogen #A11122	1;200		1;2000
Laminin 1	in house	1;500		
Paxillin	BD Biosciences #610233	1;200		
Paxillin (Y31)	Life Technologies #44720-G	1;200		
Rac1 (clone 23A8)	Merck Millipore #05-389			1;1000
Smooth muscle actin (clone 1A4)	Sigma #A2547	1;500		
Stat5a	Santa Cruz #1081	1;200		1;5000
Stat5a (Y694)	Cell Signalling Technology #9359			1;1000
Vinculin	Sigma #V4505	1;100		
ZO1 (clone R40.76)	Millipore #MAB1520	1;200		
