# Supplementary material for: Rac1 Controls Both the Secretory Function of the Mammary Gland and Its Remodeling for Successive Gestations
Source: Dev Cell. 2016 Sep 12;38(5):522–35. doi: 10.1016/j.devcel.2016.08.005 (PMC5022528; doi:10.1016/j.devcel.2016.08.005)
Supplement: Document S2. Article plus Supplemental Information [file mmc2.pdf]

Developmental Cell

Article

Rac1 Controls Both the Secretory Function of the Mammary Gland and Its Remodeling for Successive Gestations

Graphical Abstract

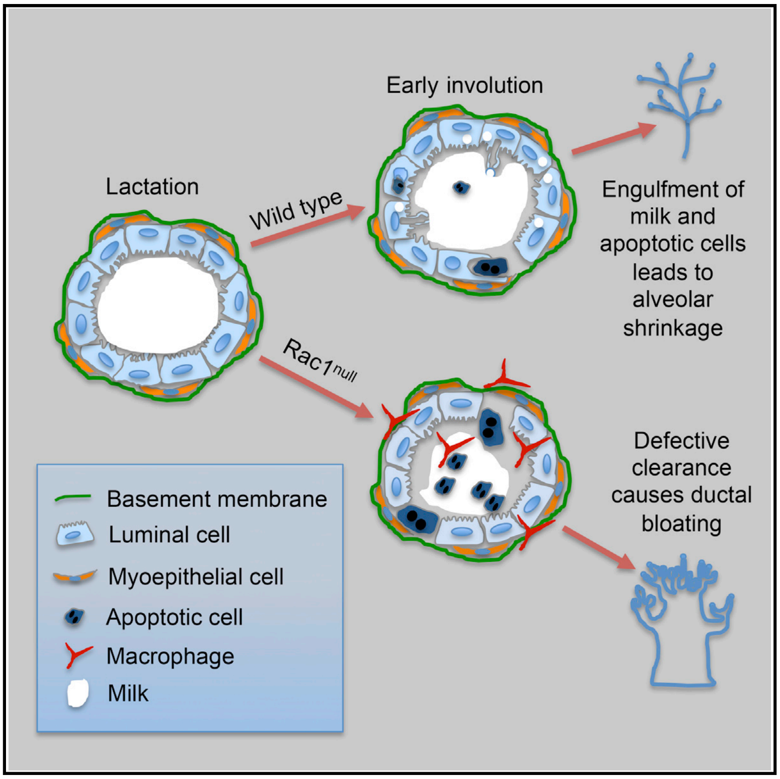

Authors

Nasreen Akhtar, Weiping Li,
Aleksander Mironov, Charles H. Streuli

Correspondence

n.akhtar@sheffield.ac.uk

In Brief

Akhtar et al. show that Rac1 gene deletion disrupts mammary gland development and causes long-term tissue malfunction. In lactation, Rac1 is required for milk production. After lactation, Rac1 causes cell function to switch from secretion to phagocytosis, thereby removing apoptotic cells and excess milk. This allows the tissue to remodel for the next pregnancy.

Highlights

- Rac1 is required for full secretory differentiation of the mammary gland
- Rac1 restricts apoptotic cell shedding into the lumen to limit inflammation
- Rac1 contributes to post-lactational tissue remodeling during involution
- Defective clearance of milk and dead cells in Rac1-null glands causes ductal bloating

Accession Numbers

E-MTAB-5019
GSE85188

Akhtar et al., 2016, Developmental Cell *38*, 522–535
September 12, 2016 © 2016 The Authors. Published by Elsevier Inc.
http://dx.doi.org/10.1016/j.devcel.2016.08.005

CellPress

Developmental Cell
Article

Rac1 Controls Both the Secretory Function of the Mammary Gland and Its Remodeling for Successive Gestations

Nasreen Akhtar,[1,2,3,*] Weiping Li,[1] Aleksander Mironov,[1] and Charles H. Streuli[1]
[1]Wellcome Trust Centre for Cell-Matrix Research, Faculty of Life Sciences, and Manchester Breast Centre, University of Manchester, Oxford Road, Manchester M13 9PT, UK
[2]Department of Oncology and Metabolism, The Bateson Centre, University of Sheffield, Beech Hill Road, Sheffield S10 2RX, UK
[3]Lead Contact
*Correspondence: n.akhtar@sheffield.ac.uk
http://dx.doi.org/10.1016/j.devcel.2016.08.005

SUMMARY

An important feature of the mammary gland is its ability to undergo repeated morphological changes during each reproductive cycle with profound tissue expansion in pregnancy and regression in involution. However, the mechanisms that determine the tissue's cyclic regenerative capacity remain elusive. We have now discovered that Cre-Lox ablation of Rac1 in mammary epithelia causes gross enlargement of the epithelial tree and defective alveolar regeneration in a second pregnancy. Architectural defects arise because loss of Rac1 disrupts clearance in involution following the first lactation. We show that Rac1 is crucial for mammary alveolar epithelia to switch from secretion to a phagocytic mode and rapidly remove dying neighbors. Moreover, Rac1 restricts the extrusion of dying cells into the lumen, thus promoting their eradication by live phagocytic neighbors while within the epithelium. Without Rac1, residual milk and cell corpses flood the ductal network, causing gross dilation, chronic inflammation, and defective future regeneration.

INTRODUCTION

The removal of surplus cells is crucial for tissue remodeling during development, and for the maintenance of organ homeostasis. One tissue that can assist in understanding the molecular basis of epithelial remodeling and tissue-specific function is the mammary gland. This tissue moderately proliferates and regresses with each menstrual cycle, but undergoes extreme expansion and regression during pregnancy, lactation, and involution. The regenerative capacity of the gland is demonstrated by its ability to undergo repeated rounds of morphogenesis in successive gestations. However, little is known about the factors that permit continued differentiation and regeneration in sequential reproductive cycles.

Involution occurs when the offspring stop suckling. It is characterized by a cessation of milk production followed by massive cell death of the secretory alveolar units, leaving the ductal tree behind (Atabai et al., 2007; Watson, 2006). While numerous studies have focused on the process of epithelial cell death, it is unclear how cell corpses are removed or how this influences subsequent tissue remodeling. The process of involution is tightly regulated in two stages (Lund et al., 1996). In the first 48 hr of mouse mammary involution, it is reversible. Here, distended secretory alveoli dedifferentiate and apoptosis becomes triggered, but re-suckling by pups can re-initiate lactation and prevent cell death. Subsequently, there is extensive cell death of the alveolar units, breakdown of the subtending basement membrane, and an influx of inflammatory phagocytes. In stage one, the numbers of professional phagocytes (macrophages) are insufficient to mediate the majority of cell clearance. So, mammary epithelial cells (MECs) switch to having a phagocytic role and clear the dead cells (Monks et al., 2005, 2008). However, the mechanism that determines this switch is not known.

Rho family GTPases control the engulfment of apoptotic cells (Hochreiter-Hufford and Ravichandran, 2013; Niedergang and Chavrier, 2005). In professional phagocytes, Rac1 is required for the cytoskeletal rearrangements that form the phagocytic cup around the target cell (Castellano et al., 2000; Miki et al., 1998). We therefore reasoned that this GTPase might have a crucial role in the epithelial cells of the involuting mammary gland. Rac1 controls the lactational differentiation of cultured MEC alveoli downstream of β1-integrin (Akhtar et al., 2009; Akhtar and Streuli, 2006; Naylor et al., 2005), and here we investigated the role of Rac1 in mammary epithelia at the genetic level. We have discovered that Rac1 dictates the secretory function of MECs during lactation, and then converts the cells to become phagocytic during the involution process. Rac1-dependent clearance of surplus milk and dead cells by mammary phagocytes is required for subsequent tissue remodeling, and for maintaining the regenerative and differentiation capacity of the mammary gland in future lactations. Our work has identified a new role for Rac1 in coordinating long-term epithelial morphogenesis and function in mammals.

RESULTS

Perinatal Lethality of Pups Nursing on Rac1 Null Mammary Glands

To examine the role of Rac1 in mammary development in vivo, we generated *Rac1*$^{fx/fx}$*:LSLYFP:WAPiCre* (*Rac1*$^{-/-}$) conditional

 © 2016 The Authors. Published by Elsevier Inc.
This is an open access article under the CC BY license (http://creativecommons.org/licenses/by/4.0/).

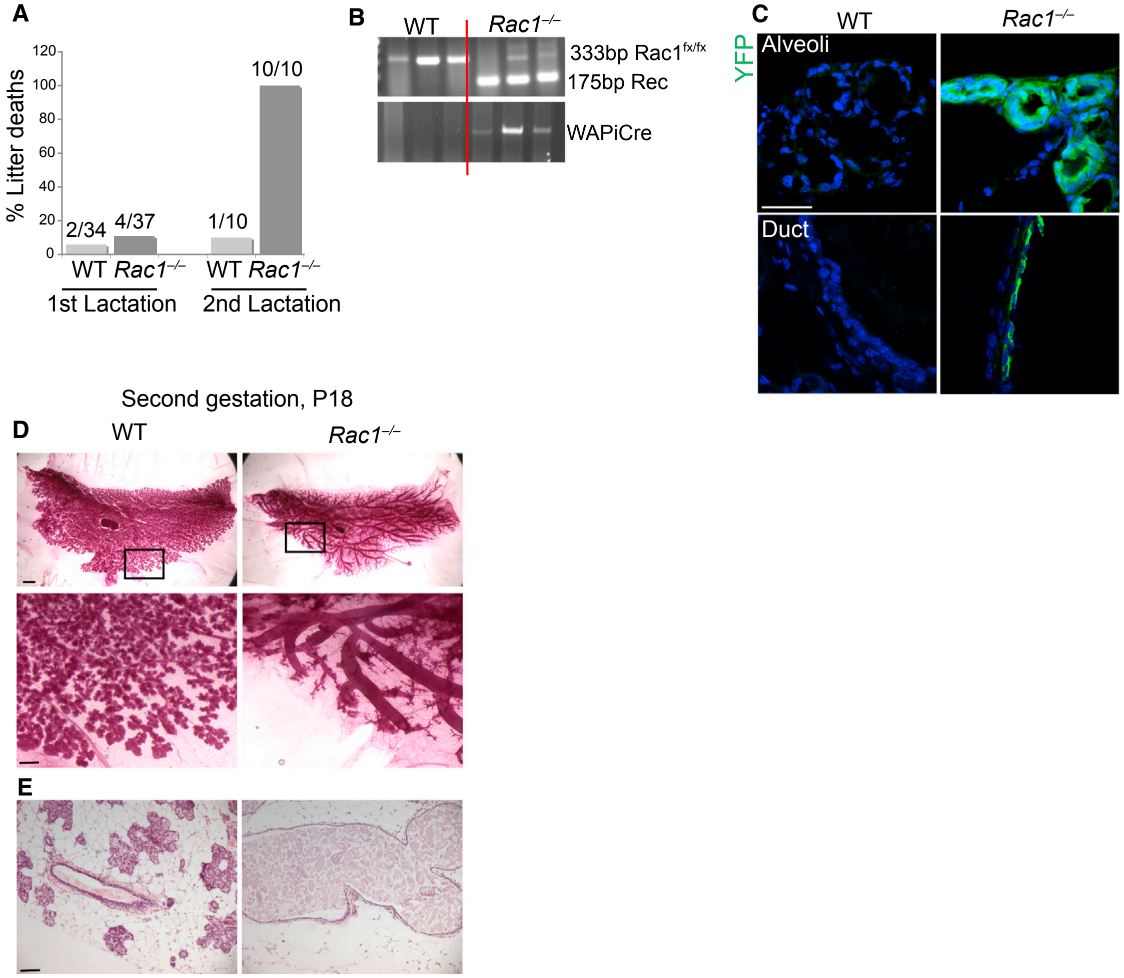

Figure 1. Loss of Rac1 Leads to Defective Alveolar and Ductal Development in Second Gestation

(A) Percentage of litter deaths at day 2 of first and second lactations.

(B) Genomic DNA was isolated from WT (*Rac1$^{fx/fx}$:LSLYFP*) and *Rac1$^{-/-}$* (*Rac1$^{fx/fx}$:LSLYFP:WAPiCre*) second-pregnancy mammary glands. PCR shows the presence of the Cre gene in luminal epithelia of transgenic glands and Cre-mediated recombination of the *Rac1$^{fx/fx}$* gene. The remaining full-length floxed allele detected in transgenics represents intact Rac1 in stromal and myoepithelial cells. The 333 bp product represents the full-length floxed allele and the 175 bp product represents the recombined *Rac1$^{-/-}$*allele. n = 3 animals are shown per group.

(C) Second-pregnancy day 18 (P18) WT and *Rac1$^{-/-}$* glands, immunostained for YFP reporter gene expression. The presence of YFP in *Rac1$^{-/-}$* glands showed that Cre-mediated recombination occurred in the luminal cells of ducts and alveoli. Bar, 45 μm.

(D) Carmine staining of whole-mounted mammary gland of *WT* and *Rac1$^{-/-}$* mice at pregnancy day 18 of the second gestation. Rac1 loss leads to ductal dilation and severe retardation of alveoli units. Bar, 2.8 mm (insert, 0.6 mm).

(E) H&E staining of mammary gland at P18, second gestation. Bar, 80 μm.

See also Figure S1.

knockout mice to delete the Rac1 gene (Akhtar and Streuli, 2013). Cre recombinase was driven by the mammary-specific whey acidic protein promoter, which is activated at mid-pregnancy in luminal epithelial cells (Figure S1). Cre-negative *Rac1$^{fx/fx}$:LSLYFP* mice were used as wild-type (WT) littermates. Most of the first litters (*Rac1$^{+/-}$* genotype) feeding on *Rac1$^{-/-}$* *transgenic* glands survived, albeit smaller in size. However, following the second gestation, all the litters died of malnourishment within 24 hr of birth, suggesting that *transgenic* dams were not able to nurse their pups (Figure 1A). Analysis of second-pregnancy glands revealed Rac1 gene deletion in *transgenics* by PCR, and loss of Rac1 in both alveoli and ducts, detected by expression of the YFP reporter gene (Figures 1B and 1C). This resulted in two major defects: impaired lobular alveolar development and gross enlargement of the mammary ducts (Figures 1D, 1E, and S2A). We named this the baobab phenotype, due to its morphological resemblance to the baobab tree. To confirm that baobab ducts were a result of Rac1 ablation and not adverse effects of Cre recombinase, we generated *WAPiCre*:*LSLYFP* mice with WT Rac1 alleles. Cre recombinase expression had no effects on ductal or alveolar morphogenesis in a second pregnancy (Figures S2B and S2C).

These data reveal key roles for Rac1 in regulating epithelial tissue fate decisions in the mammary gland. Without Rac1, the

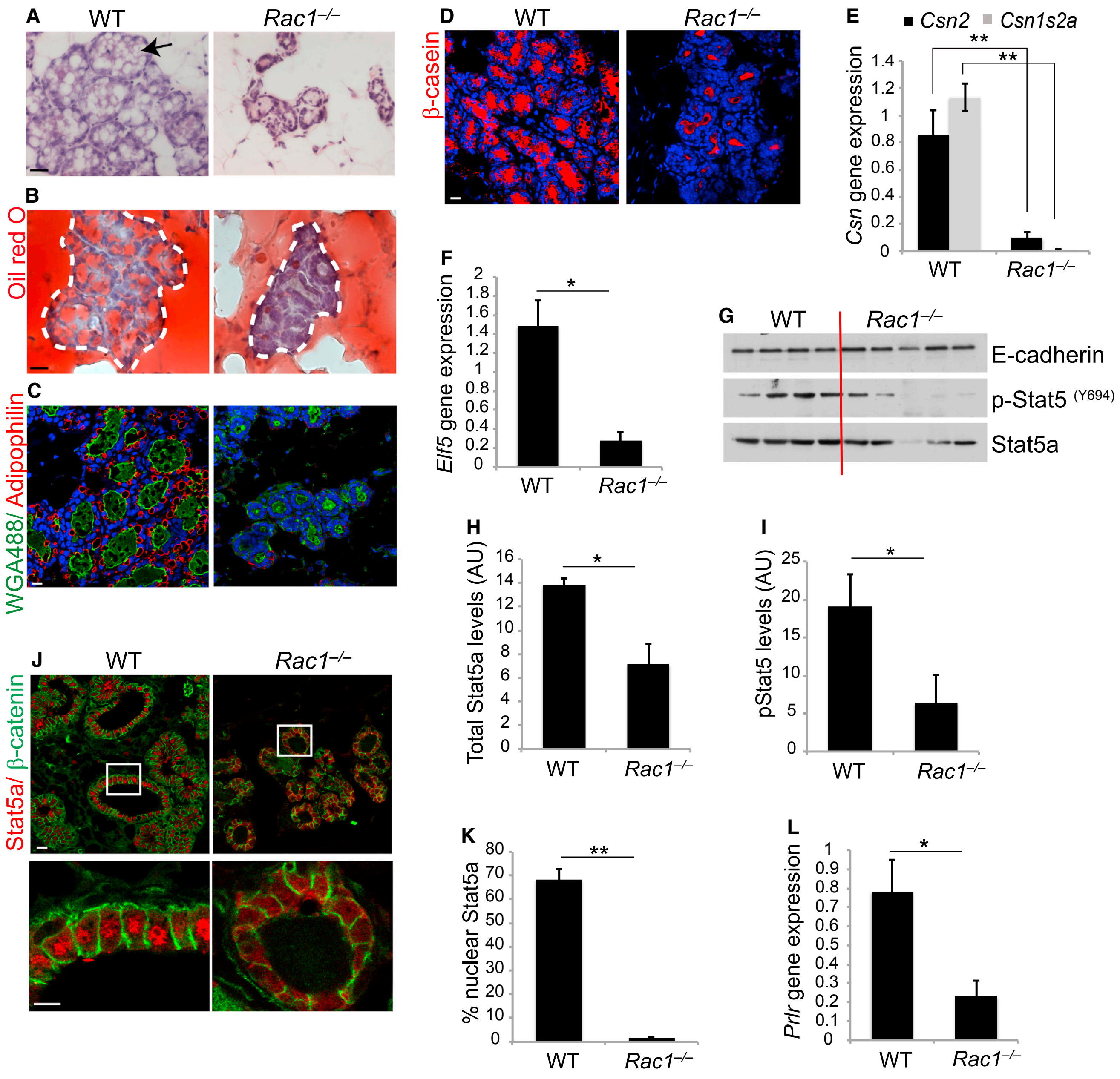

Figure 2. Second Lactation Cycle Is Severely Defective without Rac1

(A–I and L) Second gestation, P18 glands were used.

(A) H&E staining of mammary gland shows the presence of lipid droplets in WT glands (arrow). Note reduced alveolar development and an absence of lipid droplets in *Rac1*$^{-/-}$ glands. Bar, 20 μm.

(B) Oil red O staining of tissue sections, with dotted lines denoting alveolar edges. In comparison with WT, *Rac1*$^{-/-}$ glands do not contain significant quantities of milk fat in alveoli. Bar, 15 μm.

(C) Immunofluorescence for lipid envelope protein adipophilin (red) reveals large milk lipid droplets in WT glands but these are significantly reduced in *Rac1*$^{-/-}$ glands. Wheat germ agglutinin (WGA-488; green) was used to detect the luminal surface. Bar, 15 μm.

(D) Immunofluorescence staining of β-casein shows reduced milk protein in *Rac1*$^{-/-}$ glands compared with WT. Bar, 15 μm.

(E) qRT-PCR shows defective *Csn2* (β-casein) and *Csn1s2a* (γ-casein) gene expression in *Rac1*$^{-/-}$ glands. Error bars ± SEM of n = 4 mice (WT) and n = 5 mice (*Rac1*$^{-/-}$). **$p < 0.001$.

(F) qRT-PCR shows reduced Elf5 gene expression in *Rac1*$^{-/-}$ glands. Error bars ± SEM of n = 3 mice. *$p < 0.05$.

(G) Immunoblot showing expression and (Y694) phosphorylation of Stat5a. E-cadherin was used to show equal loading. WT, n = 4 mice; *Rac1*$^{-/-}$, n = 5 mice.

(H) Quantitation of the Stat5a immunoblot (G) after normalization to loading control (E-cadherin). Error bars ± SEM of n = 4 mice (WT) and n = 5 mice (*Rac1*$^{-/-}$). *$p < 0.05$.

(legend continued on next page)

epithelia preferentially switch to forming enlarged ducts rather than alveoli.

Failed Lactation in Rac1 Null Mammary Glands

To determine the possible cause of mortality in the pups feeding on *Rac1*$^{-/-}$ dams, we investigated whether lactation was altered in mammary epithelia. Where small lobular alveolar units were present, Rac1 ablation had a severe effect on the synthesis and secretion of milk fat (Figures 2A–2C). Levels of the milk proteins β- and γ-casein were also markedly reduced in *Rac1*$^{-/-}$ mammary alveoli, confirming that pups died from severe malnourishment (Figures 2D, 2E, and S3). Gene array studies revealed that, in the absence of Rac1, numerous gene sets encoding milk protein and fat synthesis were severely compromised, indicating that the alveolar secretory differentiation switch was defective (Tables S1 and S2).

One possible explanation for the lack of milk production is the reduced alveologenesis in second-pregnancy *transgenic* glands, which we investigated through several lines of enquiry. First in *transgenic* alveoli, there was reduced expression of Elf5 and Stat5 transcription factors (but not GATA3), which drive milk protein gene expression downstream of the prolactin signaling pathway (Figures 2F and 2G, Table S2). In addition, there was reduced Stat5a phosphorylation (Figures 2G–2I) and a concomitant loss in its nuclear translocation (Figures 2J and 2K), indicating a functional defect in the lactational signaling pathway. Examination of the proximal pathway components revealed a reduction in prolactin receptor expression (Figure 2L and Table S2). Second, we addressed the role of Rac1 in lactation directly, by disrupting its function after the milk-secreting alveolar units had formed. This was achieved within the first pregnancy because the WAPiCre promoter is activated following alveolar development (Figure S1). Here, the mammary ducts were not bloated, and this coincided with a lack of WAPiCre recombination in the ducts, indicating that they were still genetically WT in the first pregnancy (Figures S1 and S4A). However, *Rac1*$^{-/-}$ alveoli had reduced lumen sizes in comparison with WT alveoli, suggesting a lack of secretory products (Figures 3A and 3B). The *transgenics* also had less milk lipids and proteins (Figures 3C–3G) with a concomitant decrease in nuclear Stat5a and Elf5 gene expression (Figures S4B–D). In most cases, however, sufficient quantities of milk products were produced to support the first litter (Figure 1A).

We functionally tested the ability of *Rac1*$^{-/-}$ alveolar epithelium to synthesize β-casein when directly treated with lactogenic hormones. WT alveolar MECs form lactational acini ex vivo when embedded on a basement membrane matrix. However, when Rac1 was ablated in mammary epithelial alveolar cultures isolated from mid-pregnant *Rac1*$^{fx/fx}$*;CreER* mice, the *Rac1*$^{-/-}$ acini were compromised in milk protein synthesis (Figures 3H–3J). This confirms that the lactation defect observed in vivo is a direct consequence of Rac1 loss in the luminal MECs.

These data provide genetic evidence that Rac1 is necessary for the full lactational differentiation of mammary epithelia both in vivo and in a primary culture model.

Tissue Remodeling Defects Cause Baobab Ducts

To determine the mechanism of ductal bloating in second pregnancy, we first examined the architecture of the epithelial cells in ducts. Histological analysis revealed that luminal epithelia were typically cuboidal shaped in WT glands. In contrast *Rac1*$^{-/-}$ epithelia displayed a flattened squamous morphology, resembling myoepithelial cell architecture (Figure S5A). Mammary epithelia compartmentalize into luminal and basal (myoepithelial) cell lineages; we thus tested whether loss of Rac1 caused a lineage switch. Immunofluorescence staining with luminal and basal specific markers revealed that the flattened cells were a bilayer of both lineages positive for cytokeratin 8/18 (luminal cells), wheat germ agglutinin (luminal apical surface), and smooth muscle actin (myoepithelial cells; Figure S5B). Moreover, Rac1 loss did not affect apicobasal polarity and lineage tracing using the YFP reporter gene, which revealed that Rac1 ablation was confined to the luminal cells (Figures S5C and S5D). We functionally tested whether Rac1 controls cell height in MEC cultures from *Rac1*$^{fx/fx}$*;CreER* mice. Rac1 ablation did not affect cell height in MEC acini (Figures S5E and S5F), suggesting that ductal cell flattening in vivo was likely a result of pressure exerted by the contents of the lumen.

Examination of ductal lumens showed the presence of milk products within *Rac1*$^{-/-}$ ducts but not in WT ducts (Figures S5G and S5H), despite the reduced milk protein expression in these mice (Figures 2C–2E). This suggested possible defects in the clearance of milk from the previous gestation during post-lactational involution. Whole-mount analysis revealed that the baobab duct phenotype of *Rac1*$^{-/-}$ *transgenics* had indeed developed prior to the second pregnancy (Figure 4A). They formed in early involution and were detectable 2 days after forced weaning of the first litter, concomitant with Rac1 ablation. The bloated ducts continued to persist 4 weeks later and into the second gestation (Figures 4B, 4C, 1D, and 1E). In contrast, *Cre*^{neg}, WT littermates, and *Cre*^{pos}/*Rac1*^{wt} ducts (*WAPiCre:LSLYFP* mice) were moderately bloated in early involution but completely recovered by 4 weeks post-lactational involution (Figures 4A–4C and S5I–S5K). Further examination revealed numerous cell corpses infiltrating the luminal spaces of *Rac1*$^{-/-}$ glands, which was confirmed by cleaved caspase-3 staining (Figures 4D and 4E). These data suggested that loss of Rac1 might promote cell death. We thus functionally tested this in cells cultured from *Rac*^{fxfx}*:CreER* mice. However, the loss of Rac1 only caused a small increase in cleaved caspase-3 expression (data not shown), and when cells were induced to die through

(I) Quantitation of the p-Stat5(Y694) immunoblot (G) after normalization to normalized total Stat5a. Error bars ± SEM of n = 4 mice (WT) and n = 5 mice (*Rac1*$^{-/-}$). *$p < 0.05$.

(J) Immunofluorescence staining of Stat5a at lactation day 2 reveals reduced nuclear translocation in *Rac1*$^{-/-}$ alveoli. β-catenin was used to mark cell edges. Bar, 15 μm (insert, 7 μm).

(K) Quantitative analysis of Stat5a nuclear translocation. Nine areas/mouse were analyzed. Error bars ± SEM of n = 3 mice per group. **$p < 0.001$.

(L) qRT-PCR shows defective prolactin receptor (Prlr) gene expression in *Rac1*$^{-/-}$ glands. Error bars ± SEM of n = 3 mice. *$p < 0.05$.

See also Figure S2, Tables S1 and S2.

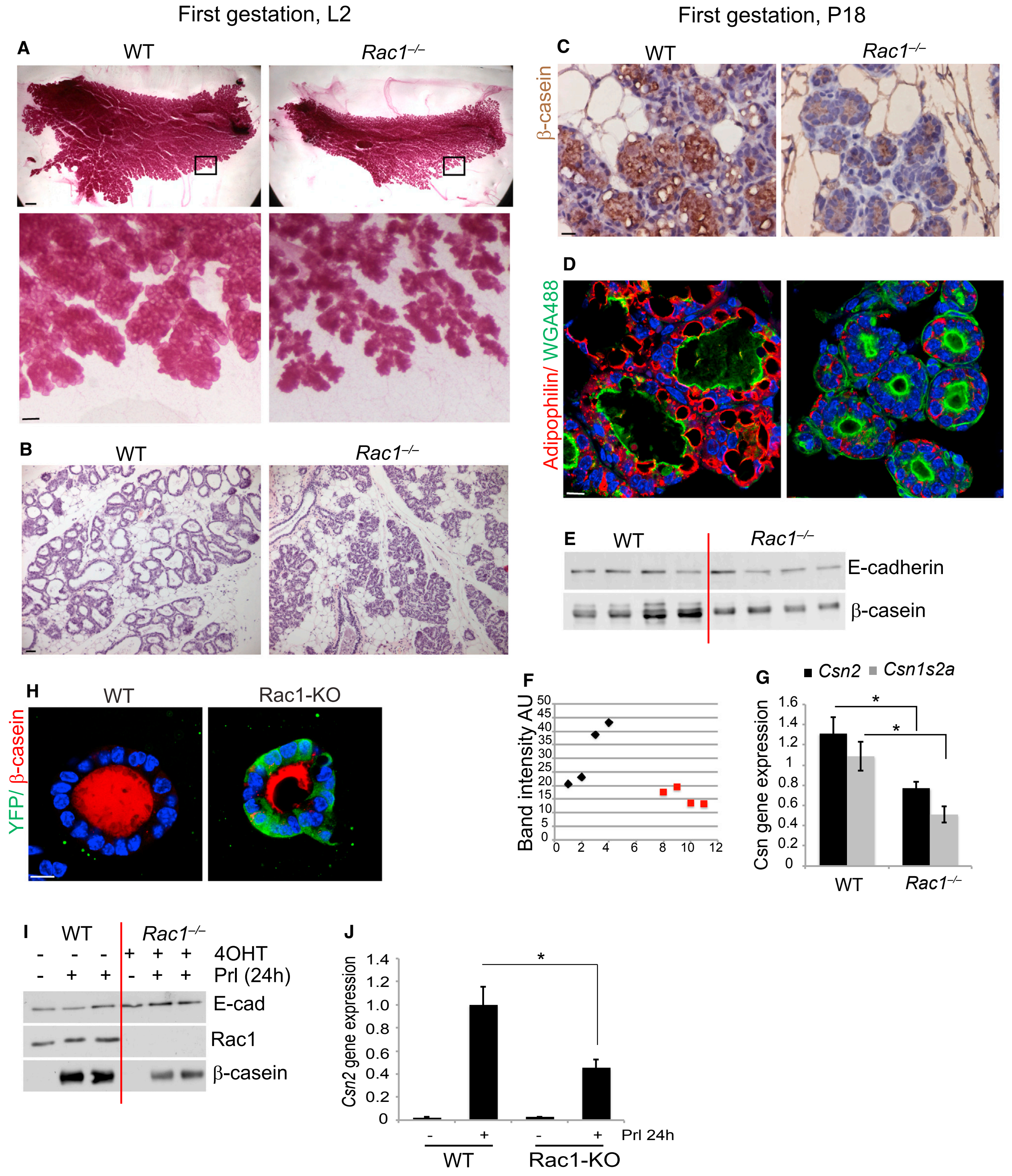

Figure 3. Loss of Rac1 from Differentiated Epithelium Results in Defective Lactation

(A) Carmine staining of whole-mounted mammary gland of *WT* and *Rac1*$^{-/-}$ mice at lactation day 2 of the first gestation. Note the smaller alveoli in *Rac1*$^{-/-}$ glands. Bar, 2.8 mm (insert, 0.33 mm).

(B) H&E staining of above mice.

(C) Immunostaining of β-casein shows reduced milk protein in P18 *Rac1*$^{-/-}$ glands compared with WT. Bar, 30 μm.

(legend continued on next page)

a loss of adhesion, there was no difference in apoptotic cell numbers (Figures 4F and 4G).

These data indicate that the baobab duct phenotype arising from the loss of Rac1 is not a result of defective epithelial architecture. Rac1 deletion does not change luminal stem cell fate into a myoepithelial lineage, and there are no gross defects in apicobasal polarity. Moreover, the accumulation of dead cells in the lumens of *transgenic* glands is not due to increased apoptosis. We therefore reasoned that the baobab phenotype might arise through either increased shedding of dying cells and/or defective phagocytic clearance of cell corpses.

Rac1 Restrains Shedding of Dying Cells from the Alveolar Epithelium

To identify a mechanism that explains cell corpse accumulation in the lumens of $Rac1^{-/-}$ glands, we tested whether loss of Rac1 promoted MEC shedding. In comparison with WT glands, where dead cells were retained within vacuolar structures associated with the alveolar epithelium, the $Rac1^{-/-}$ glands showed increased dead cell extrusion (Figures 5A and 5B). To test whether this occurred by modulating adhesion, we examined cell-cell and cell-matrix adhesions in cultured primary cells. Loss of Rac1 did not disrupt E-cadherin-mediated adhesion, indicating that altered cell-cell adhesion was an unlikely cause of increased cell extrusion (Figure 5C). In WT cells, there were numerous focal adhesions containing vinculin, paxillin, and focal adhesion kinase (FAK), with actin organized into radial bundles at the cell-ECM interface (Figures 5D, 5E, and S6A). Loss of Rac1 lowered the number of focal adhesions and reduced the organization of laminin and fibronectin (Figures 5D, 5E, S6B, and S6C). However, these cells were viable and remained attached to the ECM. A small number of focal adhesions retained the ability to initiate proximal signaling, because both FAK and paxillin were phosphorylated within adhesion complexes of $Rac1^{-/-}$ cells (Figures S6D and S6E). On trypsin treatment, $Rac1^{-/-}$ MECs detached considerably more readily than WT cells, and then failed to reattach to an ECM (Figure 5F).

Thus, Rac1 promotes MEC adhesion to the basement membrane, thereby limiting the extrusion of dying MECs from the alveolar epithelium. This proximity of cell corpses to the intact epithelium might allow easer recognition and removal by neighboring live MEC phagocytes. In contrast, without Rac1, the dying cells rapidly detach from the basement membrane, become spatially out of reach for removal by mammary phagocytes, and accumulate in luminal spaces.

Rac1 Is Critical for the Engulfment of Dead Cells

The clearance of apoptotic cell corpses and residual milk during involution can be performed by MECs as well as by immune phagocytes. Ultrastructural studies on day 2 WT involuting glands revealed the presence of apoptotic bodies inside live MECs, suggesting that engulfment of dead cells had taken place. In contrast, there was no engulfment in the $Rac1^{-/-}$ tissue. Instead, numerous necrotic cell corpses were present in the extracellular alveolar lumens (Figure 6A). We therefore tested the ability of Rac1 to mediate MEC phagocytosis of dead cells in culture. Apoptotic MECs, induced to die through suspension culture (labeled red), were added to live MEC monolayers (labeled green). Although WT MECs engulfed cell corpses in approximately 20% of cases, this number was reduced to 5% in the absence of Rac1 (Figure 6B). Internalized apoptotic bodies were detected by electron microscopy in viable WT MECs, but not in $Rac1^{-/-}$ cells (Figure 6C). Thus, Rac1 is critical for the phagocytic activity of mammary epithelia.

In the WT alveolar epithelium, extensive macropinocytic activity was detected at the apical luminal surface (Figure 6D). Numerous membrane extensions formed loops containing milk proteins, suggesting that the milk was being captured from the lumen of WT alveoli (Figures 6D and 6E). Indeed, cell-associated milk in WT epithelium was detected by immunofluorescence staining with a β-casein antibody (Figure 6G). Phagocytic cups also engulfed larger milk fat globules from the lumen (Figure 6F). In contrast, $Rac1^{-/-}$ tissues and cultured MECs completely lacked macropinocytic activity at the apical membrane (Figures 6D and 6H). Moreover, there was no evidence of milk components associated with the cells; instead, excess milk accumulated extracellularly within the luminal space (Figures 6D–6F).

Several lines of evidence suggest that the milk in involuting WT MECs is reabsorbed from the lumen. First, late pregnant tissue contained newly synthesized milk components that were secreted as smaller droplets bounded by a membrane. In comparison, the milk within involuting epithelia lacked an encapsulating membrane and was not present as discrete droplets (Figure S7A). Second, qPCR revealed virtually no new milk synthesis in involuting day 2 tissues, suggesting that milk in WT epithelia was likely engulfed from the lumen rather than newly synthesized (Figure S7B). Third, milk-engulfing macropinosomes were only detected in involuting tissue but not at the earlier secretory stage (Figure S7C). WT alveoli collapsed by involution day 4, concomitant with milk removal from the lumen (Figures 6I and 6J). In contrast, $Rac1^{-/-}$ alveoli remained distended and engorged with both milk and dead cells in the lumen.

We thus tested the ability of Rac1 to engulf milk in MEC cultures. To assess this, we used fluorescent-conjugated milk protein (FITC-casein) and measured its uptake by macropinocytosis when added to MEC cultures. Inhibition of Rac1 activity reduced uptake of FITC-casein by over 5-fold in dextran-positive

(D) Reduced milk lipid droplets revealed by adipophilin staining in P18 $Rac1^{-/-}$ glands. Wheat germ agglutinin (WGA488) was used to demark apical lumens. Bar, 50 μm.

(E) Immunoblot showing reduced β-casein in P18 $Rac1^{-/-}$ glands.

(F) β-casein immunoblot was quantified using the Odyssey imaging system (LICOR Biosciences). Black diamonds, WT glands; red squares, $Rac1^{-/-}$ glands.

(G) qRT-PCR shows defective *Csn2* and *Csn1s2a* gene expression in P18 $Rac1^{-/-}$ glands. Error bars ± SEM of n = 3 mice, $^{*}p < 0.05$.

(H) Immunofluorescence staining of β-casein and YFP in WT and Rac1-KO primary cultures. These are confocal images through the center of 3D acini cultured on a basement membrane matrix. Bar, 10 μm.

(I) Immunoblotting shows reduced β-casein levels in Rac1-KO primary cultures.

(J) qRT-PCR shows defective *Csn2* gene synthesis in response to lactogenic hormones in Rac1-KO primary cultures. Error bars ± SEM of n = 3 samples, $^{*}p < 0.05$. See also Figure S4.

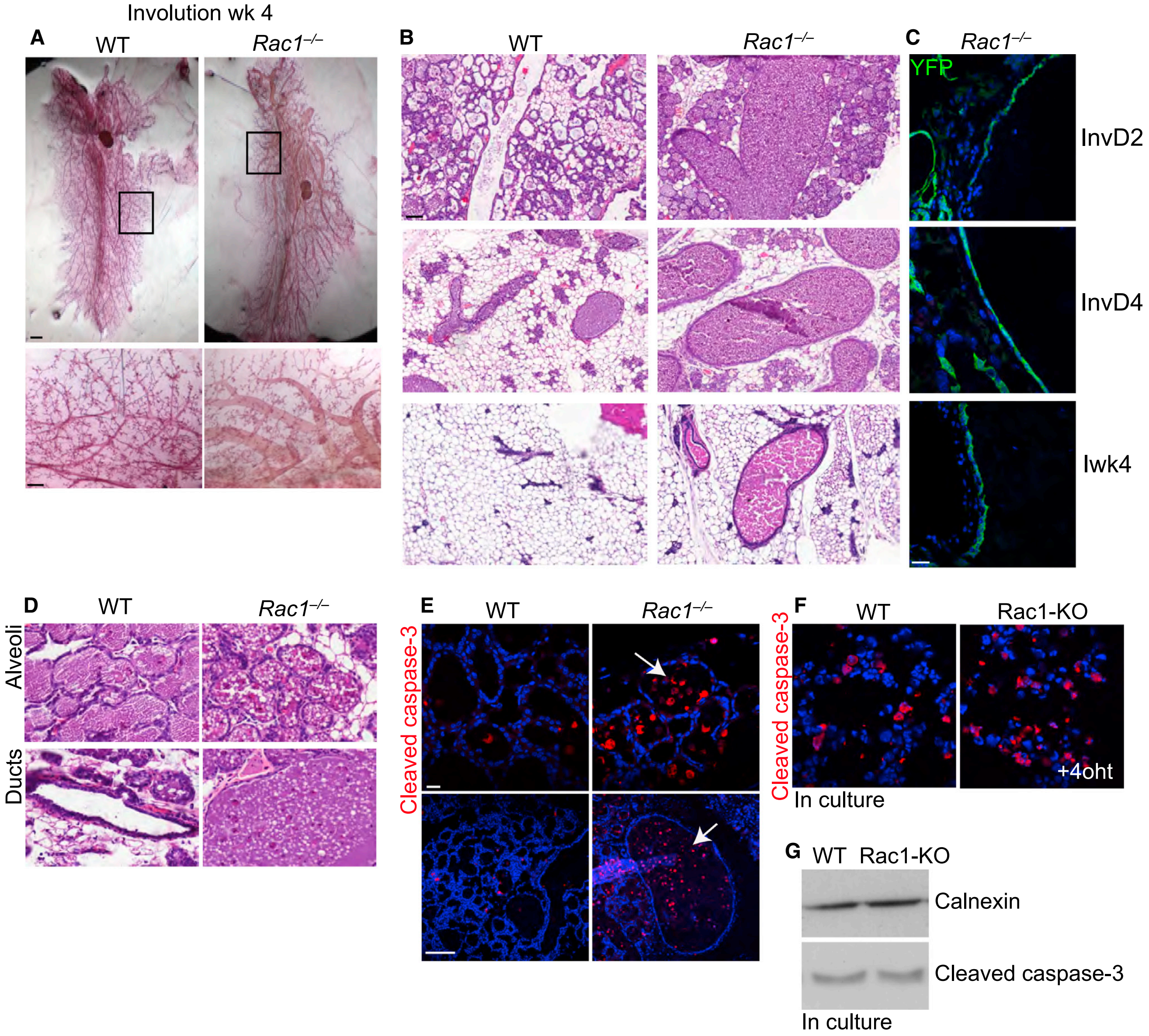

Figure 4. Baobab Ducts Arise in Post-lactational Involution, and Glandular Lumens Are Full of Dead Cells

(A) Carmine staining of whole-mounted mammary glands at 4 weeks post-lactational involution. This shows baobab ducts in *Rac1*$^{-/-}$ tissue. Bar, 2.8 mm (insert, 0.7 mm).

(B) H&E staining of WT and *Rac1*$^{-/-}$ mammary glands at involution day 2, day 4, and 4 weeks. Note *Rac1*$^{-/-}$ baobab ducts are present at involution day 2 and persist throughout involution. Bar, 200 μm.

(C) Immunofluorescence staining of YFP shows that Rac1 ablation in ducts coincides with the baobab phenotype. Bar, 45 μm.

(D) H&E stain shows accumulation of dead cells in *Rac1*$^{-/-}$ alveolar and ductal lumens.

(E) Cleaved caspase-3 staining showing dead cell accumulation in lumens of *Rac1*$^{-/-}$ glands. Arrows point to dead cells in alveoli (top panel) and ducts (bottom panel). Bar, 45 μm (top), 200 μm (bottom).

(F) Cleaved caspase-3 staining in WT and Rac1-KO primary cultures induced to die for 5 hr in suspension.

(G) Immunoblot of (F) showing no difference in caspase-3 activation in WT and Rac1-KO suspension cultures.

See also Figure S5.

macropinosomes (Figures S7D and S7E), confirming that Rac1 is required for milk clearance by MECs.

The phagocytic activity of MECs involves upregulation of surface-engulfment receptors that recognize and bind dead cells (Monks et al., 2005; Stein et al., 2004). However, the molecular switches triggering this process are not known. Gene array studies at involution day 2 revealed that, in the absence of Rac1, there was decreased expression of gene sets encoding phagocytic receptors (Figure S7F). This suggests a role for Rac1 in the priming of MECs into phagocytes in involution. An

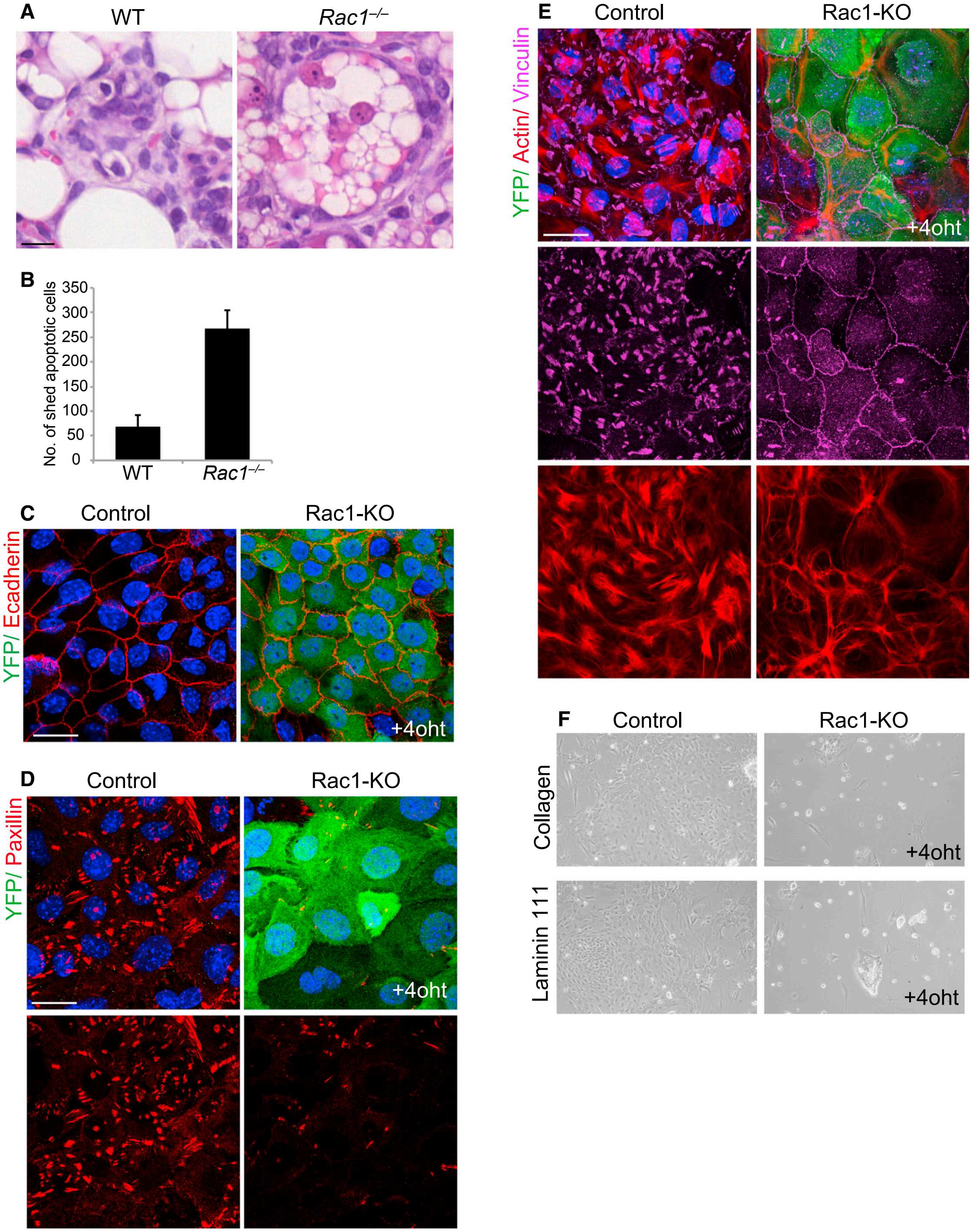

Figure 5. Reduced Cell-ECM Adhesion in *Rac1*$^{-/-}$ Epithelia Leads to Increased Cell Shedding

(A) H&E staining of WT and *Rac1*$^{-/-}$ alveoli at involution day 4. In WT glands, dying cells are contained in vacuoles within viable neighbors. In *Rac1*$^{-/-}$ glands, apoptotic cells are extruded out of the epithelium. Bar, 35 μm.

(B) Incidence of dead cell extrusion from epithelium. The number of cell corpses shed into lumens were counted in 50 alveoli per gland (involution day 2). Error bars ± SEM of n = 3 mice.

(C) E-cadherin staining shows cell-cell junctions are not disrupted following deletion of Rac1 in monolayer culture. YFP was used to detect Cre recombination.

(D) Paxillin staining shows marked reduction in focal adhesions following Rac1 deletion in culture.

(E) Vinculin and actin staining show reduced focal adhesion and distinct actin rearrangement, following Rac1 deletion.

(F) Rac1-KO cells fail to attach when re-plated onto a collagen or laminin-111 matrix.

Bar, 10 μm (C–E). See also Figure S6.

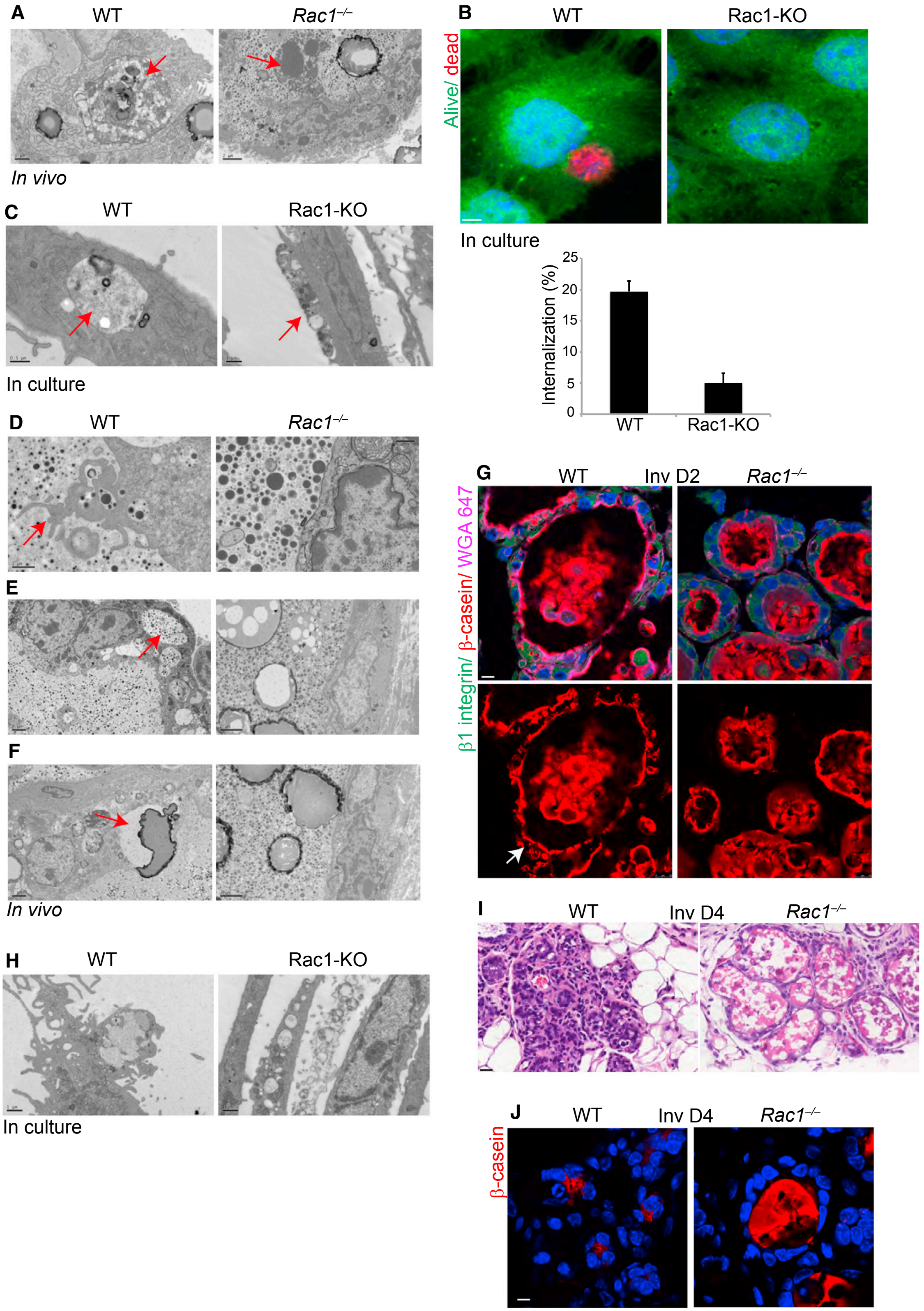

Figure 6. Rac1 Is Required for MEC Phagocytosis in Involution

(A) Electron micrographs of involution day 2 glands. Left, arrow points to a dead cell inside a live MEC in WT, also note the presence of milk fat globules. Right, arrow points to a necrotic cell in the lumen of *Rac1*$^{-/-}$ alveolus. Note, there is no engulfment in *transgenics*. Bar, 1 μm (left), 2 μm (right).

(legend continued on next page)

initial Rac1-mediated clearance may trigger a transcriptional feedback loop that further increases expression of engulfment receptors.

Together these results reveal that Rac1 is critical for both the phagocytic and macropinocytic activity of mammary epithelia. This results in the clearance of both dead cells and milk at early phases of involution. In the absence of Rac1, cell corpses and milk accumulate, eventually flooding into the adjacent ductal lumens.

Mammary Immune Responses Require Epithelial Rac1

Apoptotic cells that are not phagocytized lose contact with the basement membrane and become shed into the alveolar lumens. Ultimately, inflammatory phagocytes remove the corpses and residual milk, and thereby contribute to subsequent tissue remodeling. We therefore investigated whether the failure to clear cell corpses and milk in *Rac1*$^{-/-}$ tissue was a result of defective macrophage recruitment. In WT glands, we found no evidence of macrophages at day 2 of involution. However, they were present in the interstitial spaces at day 4 (Figure 7A). In *Rac1*$^{-/-}$ glands, there was early recruitment of macrophages at day 2 of involution, which was increased at day 4. Thus, macrophage recruitment was not defective but rather it was heightened in the absence of Rac1.

The ability of macrophages to remove dead cells and milk is dependent on their anatomical location. We therefore examined whether loss of epithelial Rac1 inhibited their entry into the lumen. In fact macrophages were detected in lumens, showing that recruitment to the correct location was not defective in *Rac1*$^{-/-}$ glands (Figure 7B). Early macrophage recruitment was associated with increased chemokine activity, detected using gene arrays; indeed there were ~20 different chemokine gene sets associated with inflammatory cell recruitment (Table S3). qRT-PCR studies detected up to 140-fold increases in chemokines CCL2 and CCL7 in some *transgenics*, which were persistently raised 4 weeks post-involution (Figures 7C and 7D). At this stage of development, immunohistochemical analysis revealed the presence of large foamy macrophages containing lipid products, confirming sustained inflammation in the *Rac1*$^{-/-}$ glands (Figures 7E and 7F).

Dying cells flip phosphatidylserine to their outer membranes, which marks them for removal by phagocytes (Fadok et al., 1992). We therefore examined whether cell corpses evaded clearance from defective exposure of phosphatidylserine. However, Rac1 ablation did not prevent phosphatidylserine exposure, detected by Annexin V binding, showing that the failure of macrophages to clear cell corpses was not due to a lack of "eat-me" recognition signals (Figure 7G).

Collectively, these data show that Rac1 modulates the cytokine and chemokine milieu of the mammary gland. Without Rac1, the inflammatory responses are both heightened and prolonged. Despite recruitment of macrophages during involution, these cells are relatively inefficient at milk clearance because residual milk fat globules persist. Rather, in the normal WT glands, the majority of milk and dead cell clearance is mediated by MECs themselves, through Rac1-dependent phagocytosis.

DISCUSSION

Our study reveals that Rac1 is a crucial mediator of the adult mammary gland developmental cycle. During lactation, Rac1 dictates the secretory function of mammary epithelia, which we now confirm with genetic analysis (Akhtar et al., 2009; Akhtar and Streuli, 2006). In involution, mammary epithelia switch their profession from secretory to phagocytic (Monks et al., 2005), which we show is also dependent on Rac1.

During involution, the mammary gland has to remove approximately 90% of its tissue weight and thus faces the burden of clearing a massive number of cell corpses. To minimize the period of inflammation, the tissue has evolved a mechanism whereby the alveolar epithelium itself clears many of the dying cells. We have now discovered that Rac1 is a key molecule that orchestrates this function. It operates at two levels to eliminate epithelial cell corpses before their entry into the lumen, thus delaying macrophage influx. First, epithelial Rac1 restricts the extrusion of dying cells from the epithelial cell layer by maintaining a small level of adhesion at the cell-basement membrane interface, and thereby promotes engulfment by neighboring live MEC phagocytes. Second, Rac1 is required for the phagocytic activity carried out by the mammary epithelia, which mediate the majority of corpse clearance.

While many of the genes associated with the involution process are under transcriptional regulation (Clarkson and Watson, 2003; Clarkson et al., 2004; Stein et al., 2004), the ability of mammary epithelia to convert rapidly from secretion to phagocytosis suggests that these cells may also switch the functions of pre-existing proteins in order to alter cell function. We propose that Rac1 mediates this. A possible mechanism enabling altered Rac1 activity is via the recruitment of distinct guanine nucleotide exchange factors (GEFs). β1-integrin is necessary for lactation through a mechanism involving the RacGEF, αPix (Akhtar et al., 2009; Akhtar and Streuli, 2006; Rooney et al., 2016).

(B) WT apoptotic MECs labeled with cell tracker red were added to WT and Rac1-KO MEC cultures, labeled with cell tracker green. Note, there is cell corpse engulfment by WT but not by Rac1-KO MECs. Bar, 3 μm. Histogram shows the percentage of cells containing engulfed apoptotic bodies.

(C) Electron micrographs of engulfment by MECs in culture as in (B). Left, arrow points to an internalized apoptotic body in live WT MEC. Right, arrow points to a necrotic cell, with no evidence of engulfment in Rac1-deficient MECs. Bar, 0.5 μm (left), 1 μm (right).

(D–F) Electron micrographs of WT and *Rac1*$^{-/-}$ involuting glands. Left panels: (D) Arrow points to macropinocytic engulfment at the apical membrane of WT MECs. Bar, 1 μm (left), 0.5 μm (right). (E) Arrow shows milk within WT MECs. (F) Arrow shows WT MEC forming a phagocytic cup around a milk fat globule. Bar, 2 μm. Right panels: Loss of Rac1 prevents all phagocytic and macropinocytic activity.

(G) Immunofluorescence staining of β-casein in WT and *Rac1*$^{-/-}$ involution day 2 tissue sections. Arrow points to cell-associated milk protein in WT glands. In *Rac1*$^{-/-}$ glands, β-casein was only detected in the lumen of alveoli and not within cells. β1-integrin and WGA-488 were used to demark cell surfaces. Bar, 7 μm.

(H) Electron micrograph showing extensive macropinocytosis in WT MEC cultures but not in Rac1 depleted cells. Bar, 1 μm.

(I) H&E staining of involution day 4 glands. Note, the alveoli have collapsed in WT glands but *Rac1*$^{-/-}$ alveoli remain engorged with milk and dead cells. Bar, 20 μm.

(J) Immunofluorescence staining of β-casein shows loss of milk from the lumen in WT glands but not *Rac1*$^{-/-}$ at involution day 4 tissues. Bar, 7 μm.

See also Figure S7.

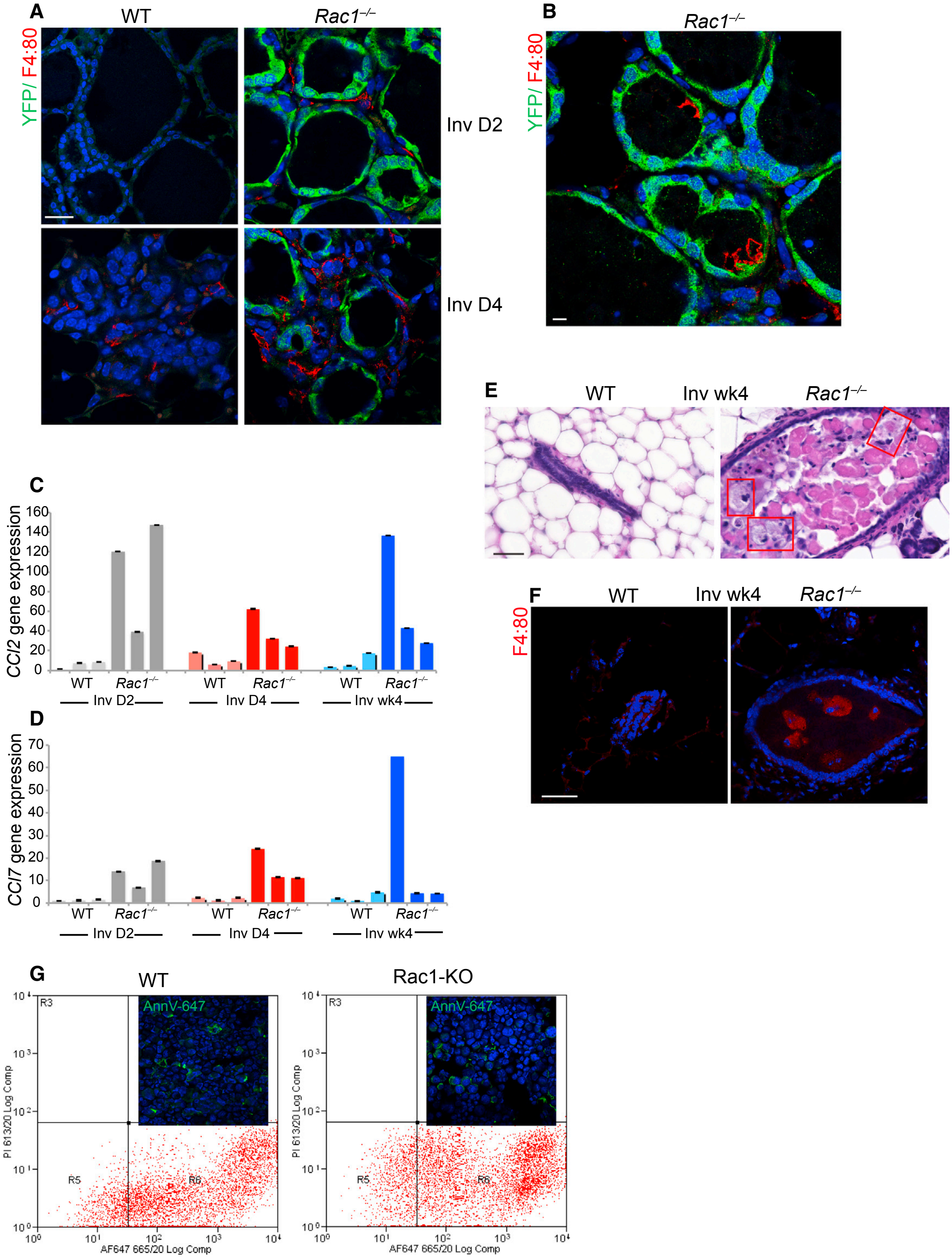

Figure 7. Loss of Rac1 Induces Chronic Inflammation

(A) Involution day 2 glands show early recruitment of F4:80 positive macrophages in *Rac1*$^{-/-}$ tissue. These were only detected in WT glands at day 4. Bar, 30 μm.

(B) Macrophages in lumen of *Rac1*$^{-/-}$ alveoli. Bar, 10 μm.

(C and D) qRT-PCR shows elevated chemokines (C) CCL2 and (D) CCL7 in *Rac1*$^{-/-}$ glands at involution day 2 (gray), day 4 (red), and 4 weeks (blue). n = 3 mice were used per group. Error bars ± SEM of triplicates taken from the same RNA sample.

(E) H&E staining of involution week 4 glands showing persistent inflammation without Rac1. Boxed areas show large foamy macrophages in duct lumens. Bar, 20 μm.

(legend continued on next page)

However, Rac1 could be regulated in post-lactational involution by separate GEFs, e.g., CrkII-Dock180-ELMO (Bagci et al., 2014; Nagata et al., 2010). Studies suggest that some of the receptors utilized for binding to dead cells are the same as in professional phagocytes (Monks et al., 2005; Stein et al., 2004). For example, loss of the bridging molecule milk fat globule epidermal growth factor (EGF) factor 8, or the Mer tyrosine kinase that specifically bind phosphatidylserine on apoptotic cells, result in reduced phagocytosis by MECs (Atabai et al., 2005; Hanayama et al., 2006; Sandahl et al., 2010). Our data suggest that Rac1 facilitates the conversion of MECs to phagocytes because, in the absence of Rac1, many of the receptors that detect dying cells fail to upregulate. Since Rac1 would be expected to function downstream of these receptors, there may be a feedback loop that further increases surface phagocytic receptor expression.

Cell corpses not removed by Rac1-deficient MEC phagocytes were shed into the lumen where they invoked inflammatory responses for removal by macrophages. Consistent with our study, ablation of Rac1 in the lung alveolar epithelium was also critical for limiting inflammatory responses (Juncadella et al., 2013). Despite an increased presence of macrophages in *Rac1*$^{-/-}$ tissue, cell corpses persisted within the luminal spaces. One possibility is that macrophages were blind to the dead cells, although the Rac1-deficient cell corpses still expressed a phosphatidylserine "eat-me" signal. Alternatively, macrophages may have become incapacitated because the burden of cell corpses simply outweighed the numbers of immune phagocytes.

Our data suggest that MEC phagocytes are also the major units responsible for removal of residual milk components and that macrophages are relatively inefficient at this process. Interestingly, engulfment of milk fat globules in a Stat3-dependent manner triggered lysosomal cell death in mammary epithelia, and without Stat3, mice have impaired involution (Kreuzaler et al., 2011; Sargeant et al., 2014). However, mammary epithelia may respond to additional death triggers, because Rac1-deficient alveoli still died despite defective engulfment. Indeed, our unpublished data suggest utilization of an apoptotic cell death pathway, suggesting the existence of multiple roads to cell removal during involution.

Milk products not removed by defective *Rac1*$^{-/-}$ MEC phagocytes rapidly flooded the interconnecting ducts, causing massive enlargement of the epithelial tree. Indeed, there is an increased proliferation of luminal progenitors in baobab ducts (unpublished data), and our future studies will focus on investigating the role of Rac1 in the maintenance of progenitor resources for alveologenesis.

The work presented here highlights the importance of MEC phagocytosis in mammalian tissue function, and it identifies a crucial role for Rac1 in the clearance of milk and cell corpses from the ductal plumbing system during involution. This is crucial for subsequent remodeling of the gland to a state that enables this tissue to develop once again into a milk-producing organ for continued mammalian reproduction.

EXPERIMENTAL PROCEDURES

Mice

The *Rac1*$^{fx/fx}$*YFP;WAPiCre*$^{Tg/\cdot}$ and *Rac1*$^{fx/fx}$*;CreER* mice were as previously described (Akhtar and Streuli, 2013). Briefly, for the in vivo analysis, Cre-mediated specific *Rac1* gene deletion in luminal MECs was achieved by crossing *Rac1*$^{fx/fx}$*RosaYFP* mice with *WAPiCre*$^{Tg/\cdot}$ mice to produce *Rac1*$^{fx/fx}$*YFP;WAPiCre*$^{Tg/\cdot}$ (*Rac1*$^{-/-}$) mice. To avoid problems in feeding of pups by mothers with potentially defective mammary glands, only the male mice of the breeding pairs carried the *Cre* transgene. *Rac1*$^{fx/fx}$*YFP* mice that lacked the *Cre* gene were used as WT controls. The genotypes of offspring were determined by PCR amplification of ear DNA as in Akhtar and Streuli (2013). To confirm gene deletion in the *Rac1*$^{-/-}$, PCR was carried out on DNA isolated from glands of each mouse used in the in vivo study. The *Rac1*$^{fx/fx}$ *RosaYFP* and *CreER* lines were crossed to produce the *Rac1*$^{fx/fx}$*YFP;CreER* (Rac1-KO) mice. These mice were used for inducible deletion of the Rac1 gene in primary cultures. *WAPiCre:LSLYFP* reporter mice were generated from *Rac1*$^{fx/fx}$*;WAPiCre*$^{Tg/\cdot}$ by breeding out with WT FVB mice to remove the floxed Rac1 alleles.

Female mice were mated between 8 and 12 weeks of age. For involution studies, dams were allowed to nurse litters (normalized to 6–8 pups) for 7–10 days, and then pups were separated from dams to initiate involution. Glands were harvested 2 days, 4 days, and 4 weeks following removal of pups. At least 6 mice per group were analyzed for each developmental stage. Mice were housed and maintained according to the University of Manchester and UK Home Office guidelines for animal research.

Primary Cell Culture and Gene Deletion

Primary MECs were harvested from 15.5- to 17.5-day pregnant mice and cultured as described in Pullan et al. (1996).

Cells were plated onto collagen 1 for monolayer cultures or basement membrane matrix (Matrigel; BD Biosciences) to form acini and cultured in growth media (Ham's F12 medium (Sigma) containing 5 μg/mL insulin, 1 μg/mL hydrocortisone (Sigma), 3 ng/mL EGF, 10% fetal calf serum (Biowittaker), 50 U/ml penicillin/streptomycin, 0.25 μg/mL fungizone, and 50 μg/mL gentamycin). Cre-mediated deletion of Rac1 in primary MEC cultures was achieved by harvesting the MECs from *Rac1*$^{fx/fx}$*;CreER* mice, and treating with 100 nM 4-hydroxytamoxifen (4OHT) dissolved in ethanol.

RNA Isolation and cDNA Synthesis

RNA was isolated from the fourth inguinal mammary gland using Trifast reagent (Peqlab) according to the manufacturer's instructions; 2 μg of RNA was used to prepare cDNA using the high-capacity RNA to cDNA kit (Invitrogen) according to the manufacturer's instructions.

qRT-PCR

qRT-PCR was performed on the Applied Biosystems 7900HT or Step One Plus system using the following Taqman probes (Applied Biosciences): *Krt18* (Mm01601704_g1), *MAPKI* (Mm00442479_m1), *Csn2* (Mm04207885_m1), *Csn1s2a* (Mm00839343_m1), *Elf5* (Mm00468732_m1), *Prlr* (Mm04336676_m1), *GATA3* (Mm00484683_m1), *CCL2* (Mm00441242_m1), *CCL7* (Mm00443113_m1). Thermal cycling conditions were as follows: UNG start at 50°C (2 min), 95°C (10 min), and then 40 cycles of 95°C (15 s) followed by annealing at 60°C (1 min).

Affymetrix Gene Array

RNA was prepared for microarray analysis using the Affymetrix Sensation kit for small quantities of low-quality-input RNA following the manufacturers' instructions. Four micrograms of the amplified, fragmented, and biotin labeled single-strand DNA fragments were applied to mouse 430 plus 2 GeneChip arrays in the appropriate hybridization cocktail and incubated at 47°C for 17 hr with rotation at 60 rpm. Following washing and staining on the fluidics

(F) Foamy macrophages in *Rac1*$^{-/-}$ duct lumens stain positive for F4:80. Bar, 20 μm.
(G) Annexin V-647^{+} cells quantified by flow sorting show no differences in phosphatidylserine exposure in WT or Rac1-deficient dying MECs. R6 = Annexin V-647^{+} fraction; WT = 83.87, Rac1-KO = 82.95. Inset shows representative micrographs of positively marked cells.
See also Table S3.

station using the Affymetrix hybridization wash and stain kit, the arrays were scanned in the GC7000 7G scanner. Initial quality control of the CEL files was carried out using the Affymetrix Expression Console software. Gene lists were analyzed using DAVID and Panther web-accessible programs. Data accession: Array Express: E-MTAB-5019 or GEO: GSE85188.

Immunostaining

Expression and distribution of various proteins were visualized by indirect immunofluorescence. Cells were fixed for 10 min in PBS/4% (w/v) paraformaldehyde, and permeabilized for 7 min using PBS/0.2% (v/v) Triton X-100. Non-specific sites were blocked with PBS/10% goat serum (1 hr, room temperature [RT]) prior to incubation with antibodies diluted in PBS/5% goat serum (1 hr, RT, each). F-actin was detected by incubating cells with TRITC-phalloidin (Sigma) for 1 hr at RT, and nuclei were stained using 4 μg/mL Hoechst 33,258 (Sigma) for 5 min at RT. Cells were washed in PBS before mounting in ProLong Gold Antifade (Molecular Probes). Images were collected on a Nikon A1 confocal or a Leica TCS SP5 AOBS inverted confocal microscope as previously described (Akhtar and Streuli, 2013). Non-biased cell counts were performed by concealing the identity of each slide.

Immunofluorescence of mammary tissue was performed on paraffin-embedded tissue (5 μm) or cryosections (10 μm), and the luminal surface was detected with wheat germ agglutinin-488, or -647 (Invitrogen, W11261, W32466) and imaged using confocal microscopy. Primary antibodies used for immunofluorescence are indicated in Supplemental Experimental Procedures. Secondary antibodies conjugated to Cy2, Rhodamine-RX, and Cy5 (Jackson ImmunoResearch).

Histology

Mammary tissue was formalin fixed (4% v/v), paraffin embedded before sectioning (5 μm), and subjected to standard H&E staining. Histology was imaged with a Zeiss Axioscop2 microscope using PlanNeoFluar 40× lens (numerical aperture 0.75) fitted with a Zeiss AxioCam color camera, and analyzed with Openlab (3.1.7) software (Improvision).

Whole-mount analysis was performed by spreading inguinal mammary glands on polysine slides and stained with carmine alum as described previously (Akhtar et al., 2009).

ORO staining was performed by staining 10 μm mammary cryosections in freshly diluted ORO solution (6 parts 0.5% ORO stock solution [Sigma] and 4 parts water) for 15 min. Sections were rinsed twice with 60% isopropanol, once with water, and then counterstained with Mayer's hematoxylin for 1 min before photography as above.

Transmission Electron Microscopy

Tissues were fixed with 4% formaldehyde + 2.5% glutaraldehyde in 0.1 M HEPES buffer (pH 7.2) for 1 hr. They were postfixed with 1% osmium tetroxide + 1.5% potassium ferrocyanide in 0.1 M cacodylate buffer (pH 7.2) for 1 hr, in 1% thiocarbohydrazide in water for 20 min, in 2% osmium tetroxide in water for 30 min, followed by 1% uranyl acetate in water overnight. The next day, they were stained with Walton lead aspartate for 1 hr at 60°C. After that, the samples were dehydrated in an ethanol series infiltrated with TAAB 812 hard-grade resin and polymerized for 24 hr at 60°C. For routine transmission electron microscopy [TEM], sections were cut with a Reichert Ultracut ultramicrotome and observed with an FEI Tecnai 12 Biotwin microscope at 100 kV accelerating voltage. Images were taken with a Gatan Orius SC1000 CCD camera.

Phagocytosis Assay

Apoptotic MECs were prepared in suspension culture overnight in serum-free media (DMEM-F12 containing 2.5 μg/mL insulin). Next day, cells were harvested by centrifugation and incubated with 5 μM Cell Tracker Red CMPTX (Invitrogen), according to the manufacturer's instructions. Live MEC monolayers (−/+4OHT) were labeled with 5 μM Cell Tracker Green CFMDA (Invitrogen) according to the manufacturer's instructions. Labeled apoptotic cells (1 × 10^6) were washed 3–4 time and then added to live MECs (2 × 10^4) for 4 hr to allow phagocytosis to proceed. Un-engulfed cells were removed by washing 5–6 times with PBS, cells were fixed in 4% paraformaldehyde, counterstained with Hoechst, and analyzed using confocal microscopy. Images were taken through the middle of the green MECs. For TEM studies, cells were unlabeled. To quantify internalization, 200 cells were counted from duplicate coverslips from three experiments and averaged to determine the percentage of cells harboring phagocytosed corpses.

Macropinocytosis Assay

To measure macropinocytosis, serum-starved Eph4 MECs grown on coverslips were stimulated with 100 ng/mL EGF to induce macropinocytosis and incubated with 100 μg/mL FITC-casein (Life Technologies), 0.5 mg/mL TRITC-Dextran 70,000 molecular weight (Life Technologies) for 15 min at 37°C. Where indicated, cells were treated with either DMSO alone or 50 μM NSC23766 Rac1 inhibitor (Stratech Scientific) overnight prior to stimulation with EGF and labeled proteins. Cells were washed once with ice-cold PBS, followed by three washes in PBS at RT and fixed in 4% paraformaldehyde, counterstained with Hoechst, and analyzed using confocal microscopy. To quantify internalization, macropinosomes containing both TRITC-Dextran and FITC-casein were counted in 100 cells from duplicate coverslips from three experiments.

Quantification of Phosphatidylserine Exposure

Apoptotic MECs (−/+4OHT) were prepared in suspension for 5 hr as above. Cells were labeled with Alexa Fluor 647-Annexin V (Biolegend) according to the manufacturer's instructions and analyzed by flow cytometry or cytospun onto polysine slides, fixed, and counterstained with Hoechst for micrographs.

Protein Analysis

Proteins were extracted as in Akhtar and Streuli (2006). Equal amounts of proteins were used and equivalent loading assessed by referral to controls, such as Calnexin (Bioquote SPA-860) or E-cadherin (Cell Signaling, 610182). Primary antibodies used for immunoblotting are indicated in Table S2. ImageJ was used to quantify bands.

ACCESSION NUMBERS

The accession number for the gene array data reported in this paper have been deposited as Array Express: E-MTAB-5019 and GEO: GSE85188.

SUPPLEMENTAL INFORMATION

Supplemental Information includes Supplemental Experimental Procedures, seven figures, and three tables and can be found with this article online at http://dx.doi.org/10.1016/j.devcel.2016.08.005.

AUTHOR CONTRIBUTIONS

N.A. conceived ideas, performed most of the experiments, and wrote the manuscript. W.L. contributed experiments, A.M. performed the electron microscopy, and C.H.S. contributed ideas and edited the manuscript.

ACKNOWLEDGMENTS

We thank Dr. Paul Heath and Dr. James Bradford for conducting the microarrays and processing the gene expression data at the Affymetrix microarray core facility and University of Sheffield. We thank the Wolfsen Light Microscopy Facility at Sheffield and the Bioimaging Facility in Manchester for use of the confocal microscopes. We thank Iwan Evans, Mark Bass, Gill Tozer, and Chryso Kanthou for critical appraisal of the manuscript. This was work was supported by the Thomas, Berry and Simpson fellowship (N.A.), the Wellcome Trust #081203/Z06/Z (C.H.S.), #200588/Z/16/Z (N.A.) and the Wellcome Trust COAF.

Received: October 20, 2015
Revised: June 30, 2016
Accepted: August 12, 2016
Published: September 12, 2016

REFERENCES

Akhtar, N., and Streuli, C.H. (2006). Rac1 links integrin-mediated adhesion to the control of lactational differentiation in mammary epithelia. J. Cell Biol. *173*, 781–793.

Akhtar, N., and Streuli, C.H. (2013). An integrin-ILK-microtubule network orients cell polarity and lumen formation in glandular epithelium. Nat. Cell Biol. *15*, 17–27.

Akhtar, N., Marlow, R., Lambert, E., Schatzmann, F., Lowe, E.T., Cheung, J., Katz, E., Li, W., Wu, C., Dedhar, S., et al. (2009). Molecular dissection of integrin signalling proteins in the control of mammary epithelial development and differentiation. Development *136*, 1019–1027.

Atabai, K., Fernandez, R., Huang, X., Ueki, I., Kline, A., Li, Y., Sadatmansoori, S., Smith-Steinhart, C., Zhu, W., Pytela, R., et al. (2005). Mfge8 is critical for mammary gland remodeling during involution. Mol. Biol. Cell *16*, 5528–5537.

Atabai, K., Sheppard, D., and Werb, Z. (2007). Roles of the innate immune system in mammary gland remodeling during involution. J. Mammary Gland Biol. Neoplasia *12*, 37–45.

Bagci, H., Laurin, M., Huber, J., Muller, W.J., and Cote, J.F. (2014). Impaired cell death and mammary gland involution in the absence of Dock1 and Rac1 signaling. Cell Death Dis. *5*, e1375.

Castellano, F., Montcourrier, P., and Chavrier, P. (2000). Membrane recruitment of Rac1 triggers phagocytosis. J. Cell Sci. *113*, 2955–2961.

Clarkson, R.W., and Watson, C.J. (2003). Microarray analysis of the involution switch. J. Mammary Gland Biol. Neoplasia *8*, 309–319.

Clarkson, R.W., Wayland, M.T., Lee, J., Freeman, T., and Watson, C.J. (2004). Gene expression profiling of mammary gland development reveals putative roles for death receptors and immune mediators in post-lactational regression. Breast Cancer Res. *6*, R92–R109.

Fadok, V.A., Voelker, D.R., Campbell, P.A., Cohen, J.J., Bratton, D.L., and Henson, P.M. (1992). Exposure of phosphatidylserine on the surface of apoptotic lymphocytes triggers specific recognition and removal by macrophages. J. Immunol. *148*, 2207–2216.

Hanayama, R., Miyasaka, K., Nakaya, M., and Nagata, S. (2006). MFG-E8-dependent clearance of apoptotic cells, and autoimmunity caused by its failure. Curr. Dir. Autoimmun. *9*, 162–172.

Hochreiter-Hufford, A., and Ravichandran, K.S. (2013). Clearing the dead: apoptotic cell sensing, recognition, engulfment, and digestion. Cold Spring Harb. Perspect. Biol. *5*, a008748.

Juncadella, I.J., Kadl, A., Sharma, A.K., Shim, Y.M., Hochreiter-Hufford, A., Borish, L., and Ravichandran, K.S. (2013). Apoptotic cell clearance by bronchial epithelial cells critically influences airway inflammation. Nature *493*, 547–551.

Kreuzaler, P.A., Staniszewska, A.D., Li, W., Omidvar, N., Kedjouar, B., Turkson, J., Poli, V., Flavell, R.A., Clarkson, R.W., and Watson, C.J. (2011). Stat3 controls lysosomal-mediated cell death in vivo. Nat. Cell Biol. *13*, 303–309.

Lund, L.R., Romer, J., Thomasset, N., Solberg, H., Pyke, C., Bissell, M.J., Dano, K., and Werb, Z. (1996). Two distinct phases of apoptosis in mammary gland involution: proteinase-independent and -dependent pathways. Development *122*, 181–193.

Miki, H., Suetsugu, S., and Takenawa, T. (1998). WAVE, a novel WASP-family protein involved in actin reorganization induced by Rac. EMBO J. *17*, 6932–6941.

Monks, J., Rosner, D., Geske, F.J., Lehman, L., Hanson, L., Neville, M.C., and Fadok, V.A. (2005). Epithelial cells as phagocytes: apoptotic epithelial cells are engulfed by mammary alveolar epithelial cells and repress inflammatory mediator release. Cell Death Differ. *12*, 107–114.

Monks, J., Smith-Steinhart, C., Kruk, E.R., Fadok, V.A., and Henson, P.M. (2008). Epithelial cells remove apoptotic epithelial cells during post-lactation involution of the mouse mammary gland. Biol. Reprod. *78*, 586–594.

Nagata, S., Hanayama, R., and Kawane, K. (2010). Autoimmunity and the clearance of dead cells. Cell *140*, 619–630.

Naylor, M.J., Li, N., Cheung, J., Lowe, E.T., Lambert, E., Marlow, R., Wang, P., Schatzmann, F., Wintermantel, T., Schuetz, G., et al. (2005). Ablation of beta1 integrin in mammary epithelium reveals a key role for integrin in glandular morphogenesis and differentiation. J. Cell Biol. *171*, 717–728.

Niedergang, F., and Chavrier, P. (2005). Regulation of phagocytosis by Rho GTPases. Curr. Top. Microbiol. Immunol. *291*, 43–60.

Pullan, S., Wilson, J., Metcalfe, A., Edwards, G.M., Goberdhan, N., Tilly, J., Hickman, J.A., Dive, C., and Streuli, C.H. (1996). Requirement of basement membrane for the suppression of programmed cell death in mammary epithelium. J. Cell Sci. *109*, 631–642.

Rooney, N., Wang, P., Brennan, K., Gilmore, A.P., and Streuli, C.H. (2016). The integrin-mediated ILK-Parvin-alphaPix signalling Axis controls differentiation in mammary epithelial cells. J. Cell. Physiol. *231*, 2408–2417.

Sandahl, M., Hunter, D.M., Strunk, K.E., Earp, H.S., and Cook, R.S. (2010). Epithelial cell-directed efferocytosis in the post-partum mammary gland is necessary for tissue homeostasis and future lactation. BMC Dev. Biol. *10*, 122.

Sargeant, T.J., Lloyd-Lewis, B., Resemann, H.K., Ramos-Montoya, A., Skepper, J., and Watson, C.J. (2014). Stat3 controls cell death during mammary gland involution by regulating uptake of milk fat globules and lysosomal membrane permeabilization. Nat. Cell Biol. *16*, 1057–1068.

Stein, T., Morris, J.S., Davies, C.R., Weber-Hall, S.J., Duffy, M.A., Heath, V.J., Bell, A.K., Ferrier, R.K., Sandilands, G.P., and Gusterson, B.A. (2004). Involution of the mouse mammary gland is associated with an immune cascade and an acute-phase response, involving LBP, CD14 and STAT3. Breast Cancer Res. *6*, R75–R91.

Watson, C.J. (2006). Involution: apoptosis and tissue remodelling that convert the mammary gland from milk factory to a quiescent organ. Breast Cancer Res. *8*, 203.

Developmental Cell, Volume *38*

Supplemental Information

Rac1 Controls Both the Secretory Function of the Mammary Gland and Its Remodeling for Successive Gestations

Nasreen Akhtar, Weiping Li, Aleksander Mironov, and Charles H. Streuli

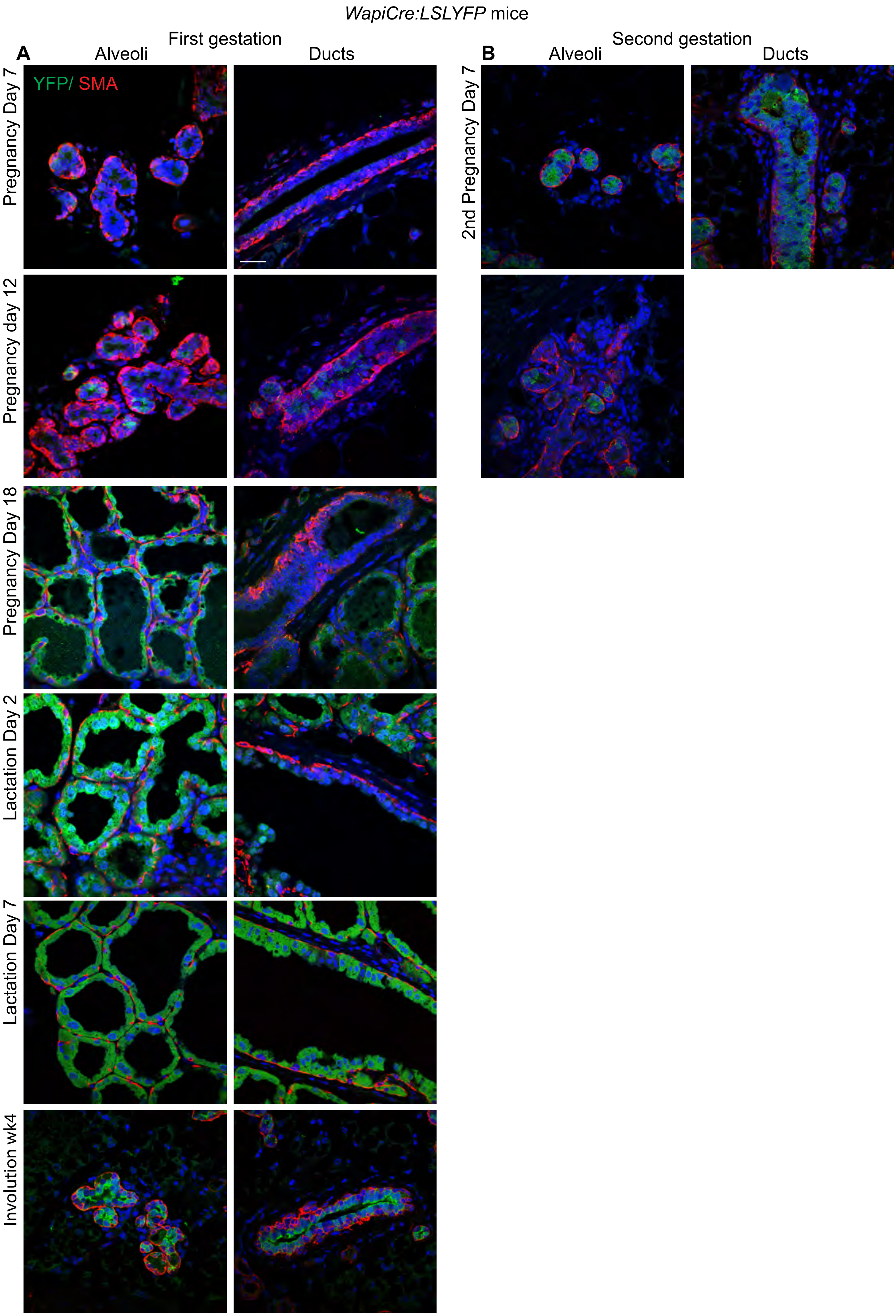
WapiCre:LSLYFP mice
First gestation
A
Alveoli
Ducts
YFP/ SMA
Pregnancy Day 7
Pregnancy day 12
Pregnancy Day 18
Lactation Day 2
Lactation Day 7
Involution wk4
Second gestation
B
Alveoli
Ducts
2nd Pregnancy Day 7

Figure S1

Figure S1 related to Fig 1-3: *WAPiCre mediated recombination during different stages of mammary gland development.*

WAPiCre mediated recombination was detected in the mammary gland at various stages of pregnancy and lactation in the first and second cycle, using a YFP reporter transgenic mouse line (*WAPiCre:LSLYFP*).

(A) GFP immunostaining was used to detect YFP reporter gene expression in alveoli (left panel) and ducts (right panel) in the first cycle at pregnancy (P) days 7, 12, 18, lactation day 2, 7 and 4 weeks post-lactational involution. Smooth muscle actin (SMA) immunostaining was used to detect myoepithelia. Extensive *WAPiCre* mediated recombination was detected within alveoli in late pregnancy (P18) after alveologenesis had proceeded and within ducts around lactation day 7. YFP reporter gene expression was still evident in glands 4 weeks post-lactational involution.

(B) In the second cycle, new alveoli detected at P7 arise from stem/progenitors that have undergone recombination in the first cycle (top left, YFP positive) but also from stem/progenitor populations that escaped recombination in the first cycle (bottom panel, absence of YFP in 20-30% of the gland).

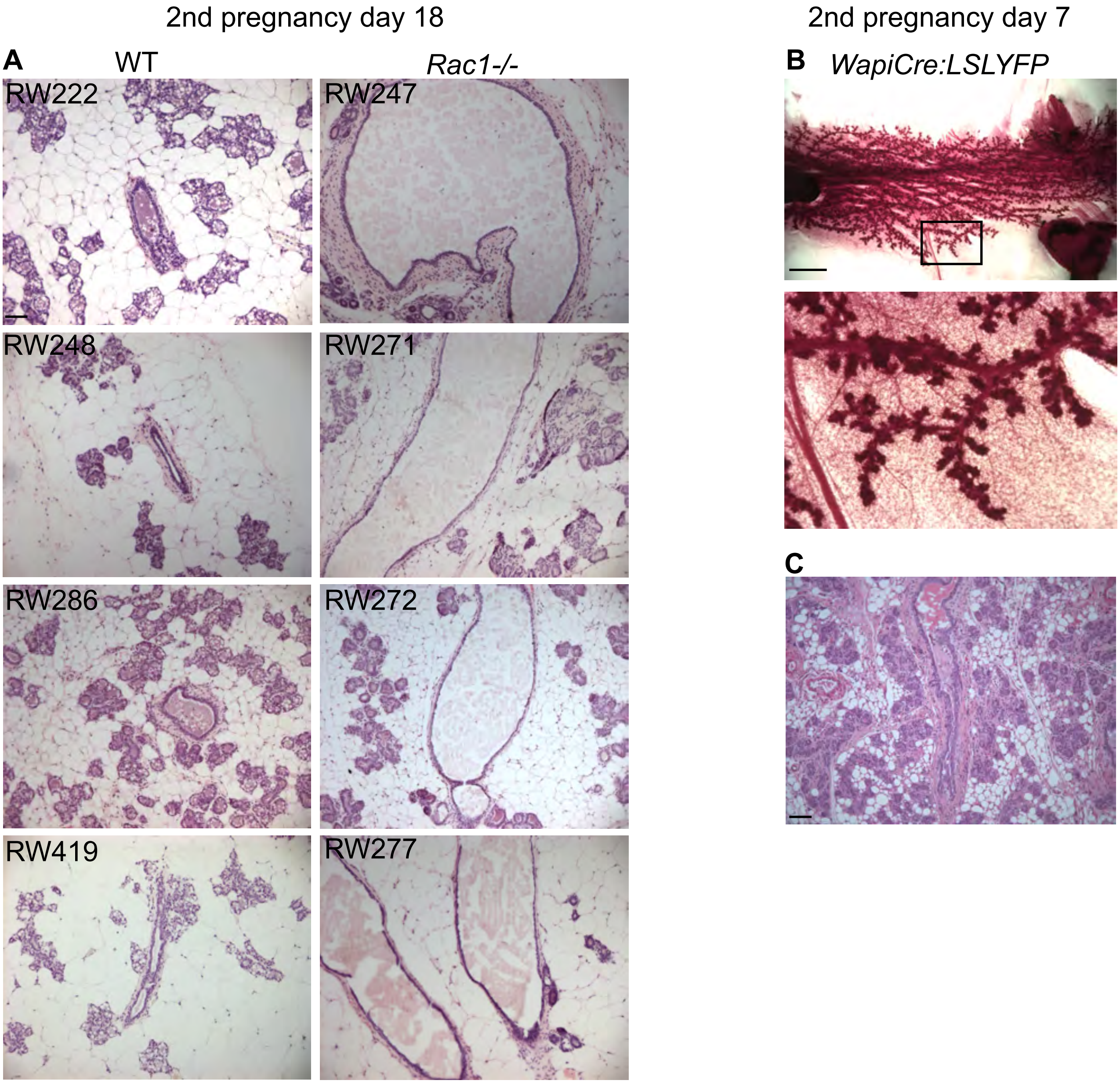

Figure S2 related to Fig 1: *Baobab duct phenotype develops specifically in Rac1-deficient mammary glands.*
(A) Representative Haematoxylin and Eosin stains of n=4 mice showing gross dilation of mammary ducts in day 18 of a second pregnancy. Bar: 100μm. RW (RacWAP) numbers = mouse identification in colony.
(B) Carmine staining of wholemounted *WAPiCre:LSLYFP* glands expressing WT Rac1 alleles show normal alveolar and ductal morphogenesis in a second pregnancy. Day 7 of pregnancy were used to visualise mammary ducts more clearly. Bar: 5mm.
(C) Haematoxylin and Eosin stain of glands as in (B). Bar: 100μm.

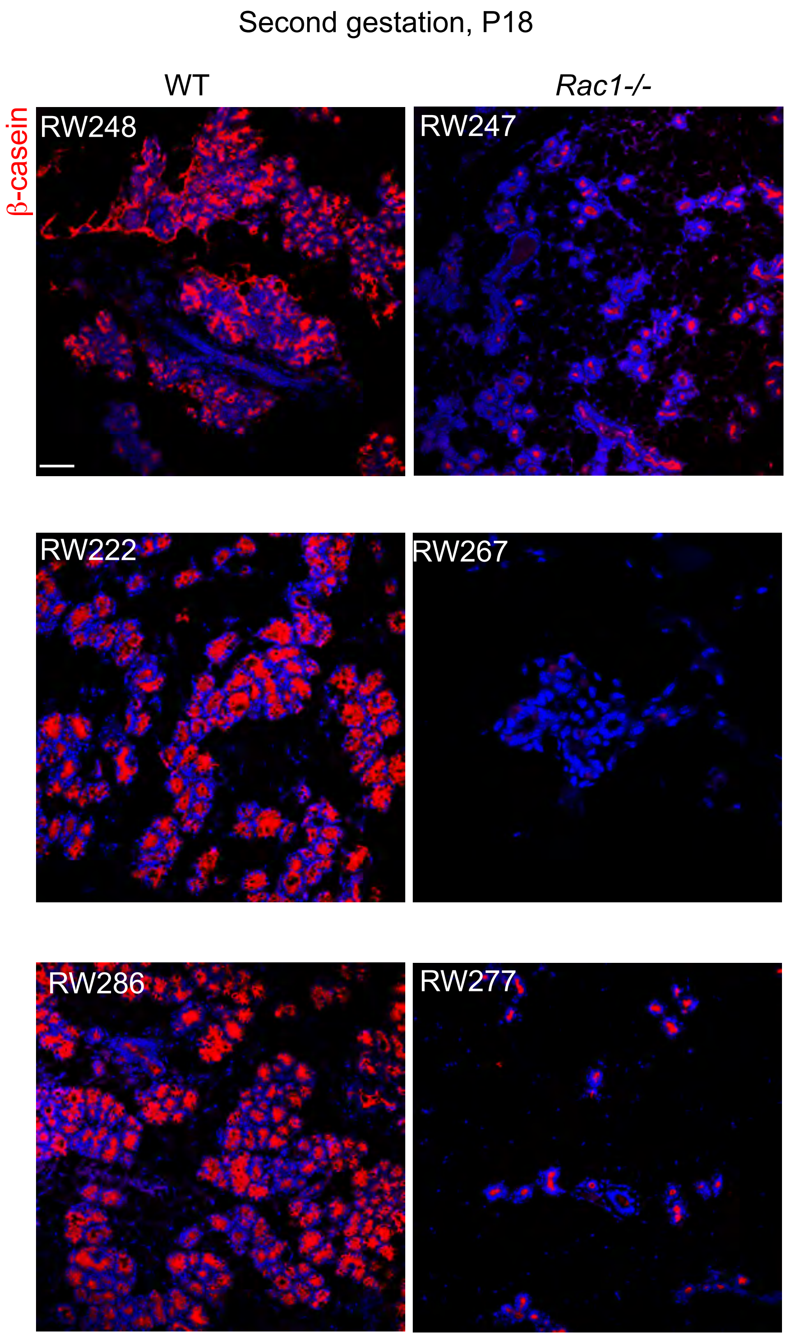

Figure S3 related to Fig 2: *Lactation defect in Rac1-/- mammary glands.*
Representative β-casein immunofluorescence stains of n=3 mice showing defective lactation at day 18 of a second pregnancy. Earclip numbers are indicated on micrographs. Bar: 150µm.

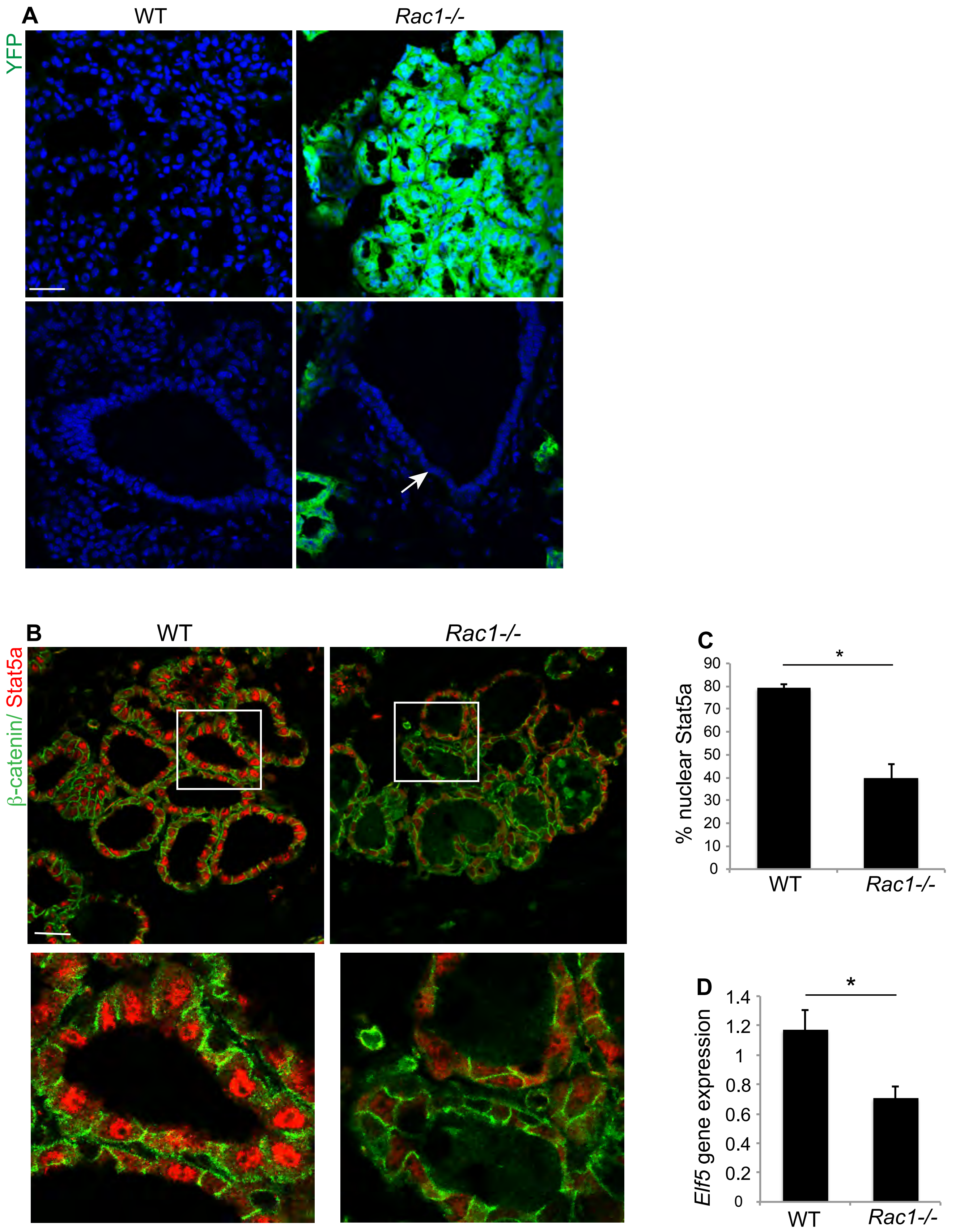
A
WT
Rac1-/-
YFP
B
WT
Rac1-/-
β-catenin/ Stat5a
C
*
% nuclear Stat5a
90
80
70
60
50
40
30
20
10
0
WT
Rac1-/-
D
*
Elf5 gene expression
1.4
1.2
1
0.8
0.6
0.4
0.2
0
WT
Rac1-/-

Figure S4

Figure S4 related to Fig 3: *Rac1 ablation and milk transcription factor activity in the first cycle.*
A) P18 WT and *Rac1-/-* glands, immuno-stained for YFP reporter gene expression. The presence of YFP in *Rac1-/-* glands showed that Cre-mediated recombination occurred in alveoli but not ducts. Arrow points to a genetically WT duct in *transgenic* tissue. Bar: 45mm.
(B) Immunofluorescence staining of Stat5a at lactation day 2 reveals reduced nuclear translocation in *Rac1-/-* alveoli. b-catenin was used to mark cell edges. Bar: 15mm (insert: 7mm).
(C) Quantitative analysis of Stat5a nuclear translocation. Nine areas/mouse were analysed. Error bars; +/- SEM of n=3 mice per group. *P=<0.05.
(D) Quantitative RT-PCR shows reduced Elf5 gene expression in P18 *Rac1-/-* glands. Error bars: +/- SEM of n=3 mice. * P<0.05

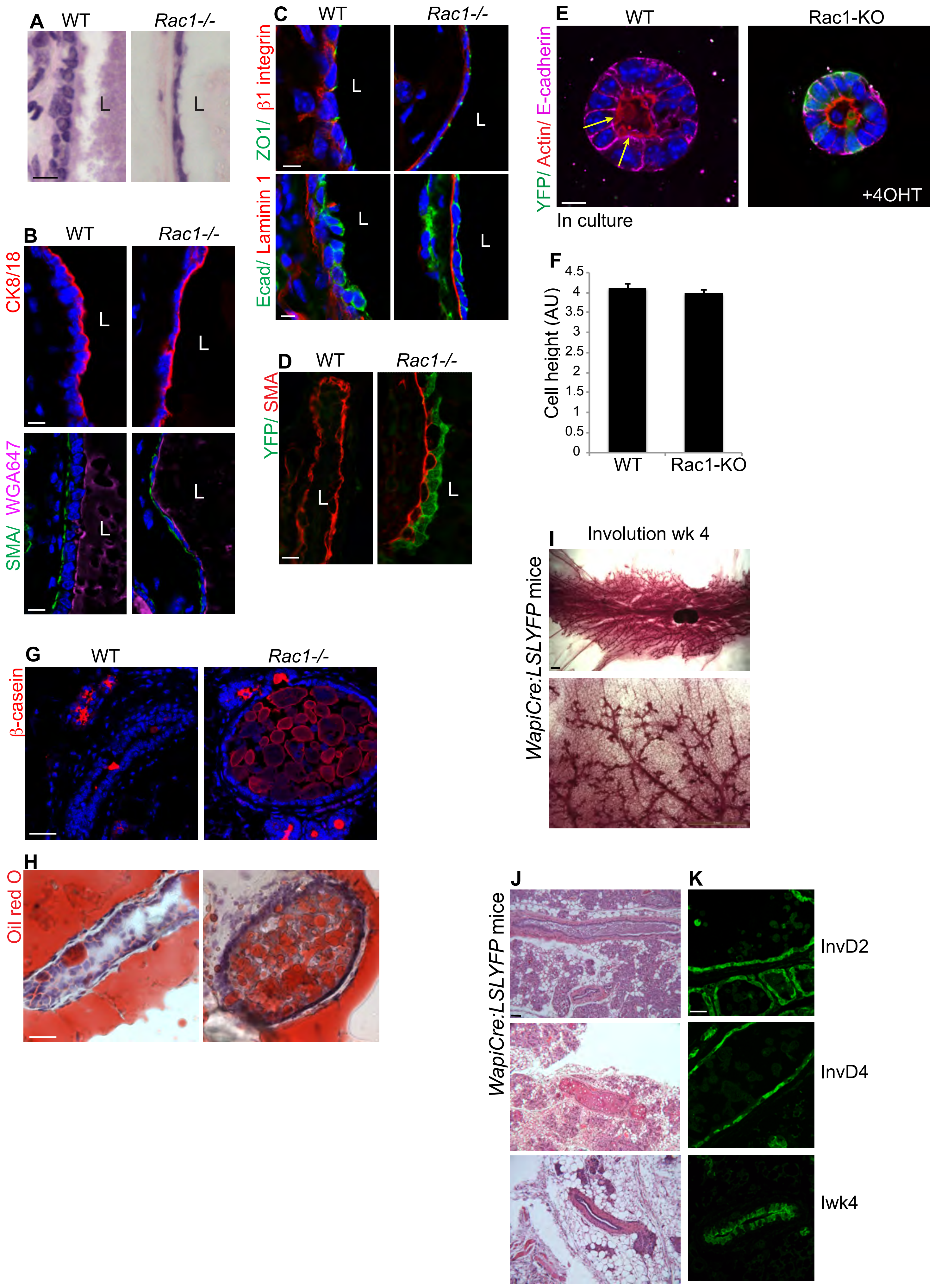
A WT Rac1-/-
L
B WT Rac1-/-
CK8/18
SMA/ WGA647
C WT Rac1-/-
ZO1/ β1 integrin
Ecad/ Laminin 1
D WT Rac1-/-
YFP/ SMA
E WT Rac1-KO
YFP/ Actin/ E-cadherin
+4OHT
In culture
F
Cell height (AU)
4.5
4
3.5
3
2.5
2
1.5
1
0.5
0
WT
Rac1-KO
G WT Rac1-/-
β-casein
H
Oil red O
I
Involution wk 4
WapiCre:LSLYFP mice
J
K
WapiCre:LSLYFP mice
InvD2
InvD4
Iwk4

Figure S5

Figure S5 related to Fig 4: *Characterisation of epithelial architecture in baobab ducts.*
(A) Haematoxylin and Eosin stain shows that *Rac1-/-* baobab ducts exhibit flattened epithelia.
(B) Baobab ducts are composed of a bi-layer of luminal and myoepithelial cells. Keratin 8/18 and WGA-488 were used to detect luminal cells and smooth muscle actin (SMA) to detect myoepithelia.
(C) Apicobasal polarity is still intact in *Rac1-/-* baobab ducts.
(D) YFP reporter gene expression shows that Rac1 ablation is restricted to luminal epithelia in baobab ducts.
(E) Representative confocal image of WT and Rac1-KO acini used to measure cell height. Actin and E-cadherin was used to demark cell edges. Bar; 10mm.
(F) Quantification of cell height in WT and Rac1-KO acini. Error bars; +/-SEM of 99 cell heights per condition.
(G) Immunofluorescence stain with b-casein showing milk products in baobab duct lumens.
(H) Oil red O stain showing accumulation of milk fat globules in baobab duct lumens.
L= lumen. Bar: (a-d) 5mm, (e) 10mm, (g,h) 40mm.
(I) Carmine staining of whole-mounted mammary glands from mice at 4 weeks post-lactational involution shows no bloating of ducts. Bar: 2.8 mm.
(J) H+E stain of mammary glands at involution day 2,4 and 4 weeks. Bar: 100mm.
(K) Immunofluorescence staining of YFP shows efficient WAPiCre mediated recombination in *WAPiCre:LSLYFP* mice. Bar: 45mm.

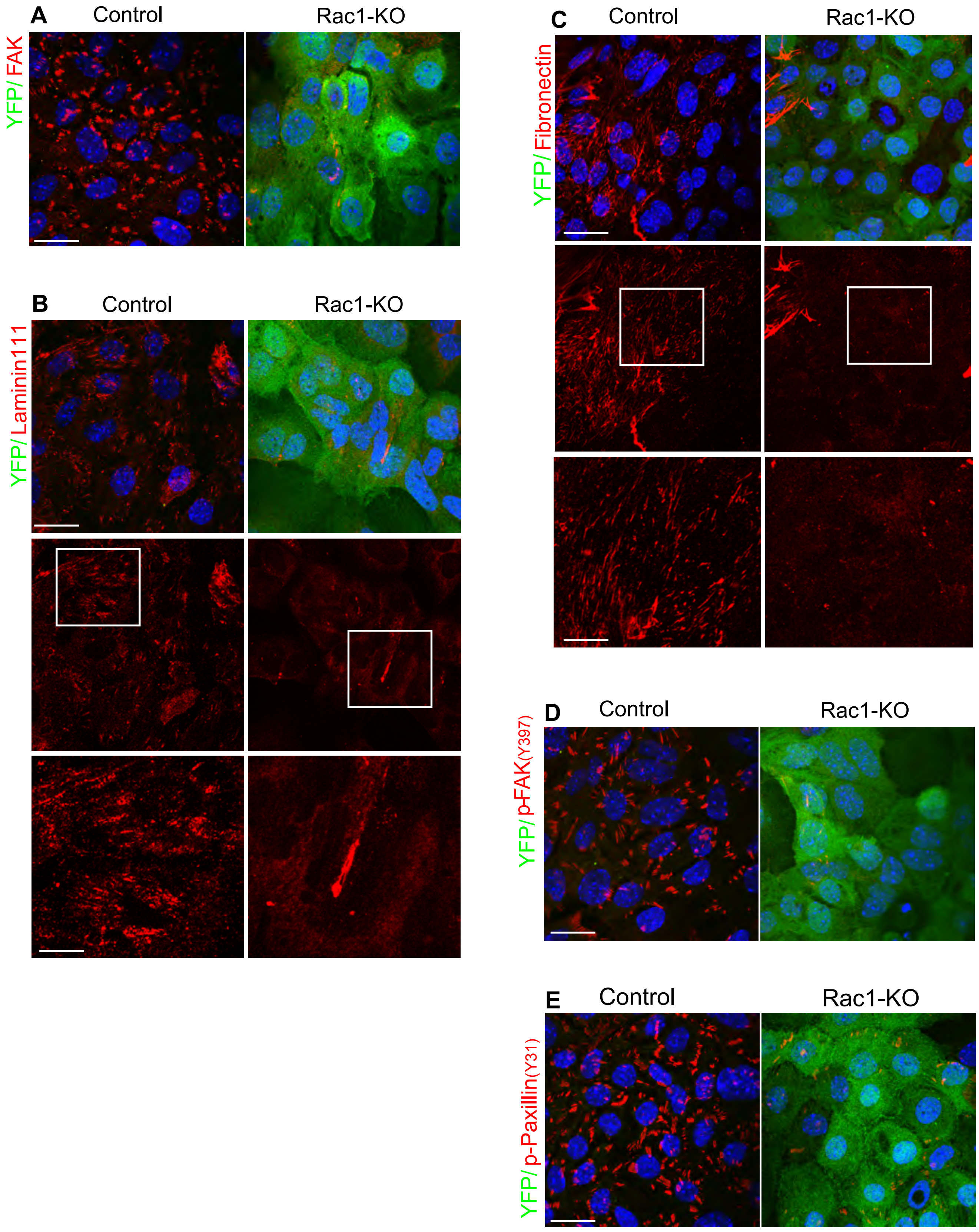

Figure S6 related to Fig 5: *Reduced cell-ECM adhesion in Rac1 depleted MECs.*
(A-E) GFP antibody stain was used to detect YFP expression in Rac1-KO MECs.
(A) Immunostaining with FAK shows markedly reduced focal adhesion complexes in Rac1-KO MECs in culture.
(B) Reduced laminin111 organisation within focal adhesions in Rac1-KO MECs.
(C) Reduced fibronectin organisation within focal adhesions in Rac1-KO MECs.
(D) Immunofluorescence stain with phosphorylated FAK (Y397).
(E) Immunofluorescence stain with phosphorylated paxillin (Y31).
Bar: 10µm, (inset 4µm).

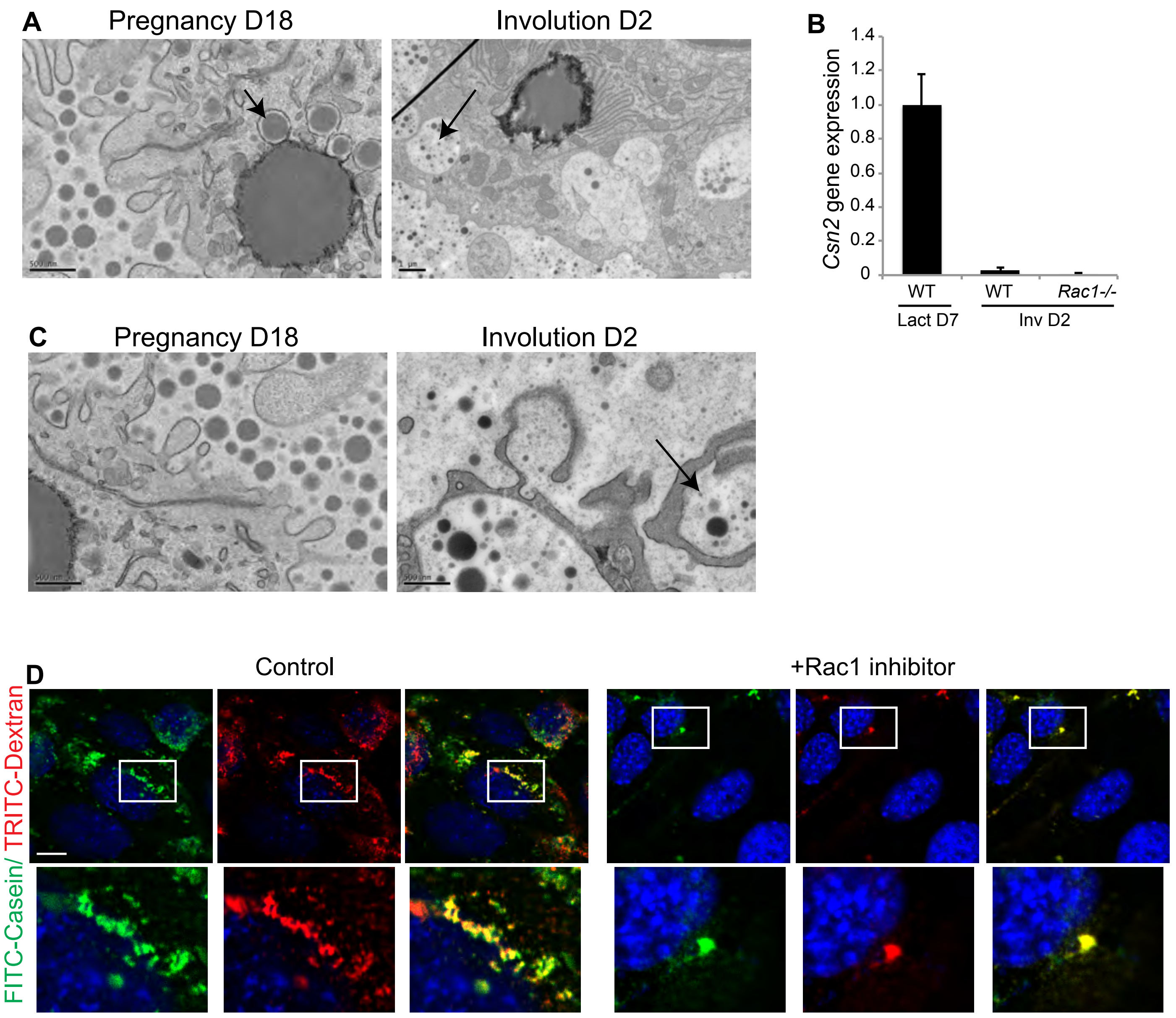

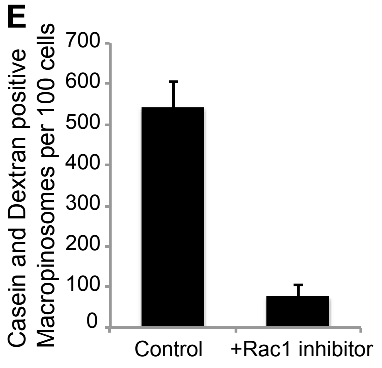

F

Gene	Fold change	P-Value
TYRO3 protein tyrosine kinase 3	-4.03	6.27E-04
CD36 antigen	-3.12	1.42E-02
CD14 antigen	-2.16	1.10E-01
milk fat globule-EGF factor 8 protein	-2.35	1.17E-01
AXL receptor tyrosine kinase	-1.30	2.88E-01

Figure S7 related to Fig 6: *Impaired engulfment in the absence of Rac1.*
(A) Electron micrograph of *left;* pregnancy day 18 and *right;* involution day 2 mammary glands. Arrows points to milk micelles in the cell. Note; milk micelles before secretion are encapsulated with a membrane (left). Bar: *left*; 500nm and *right*; 1mm.
(B) Quantitative RT-PCR for *Csn2* gene expression, showing virtually no new milk synthesis at involution day 2, compared with Lactation day 7. Error bars; +/-SEM of n=3 mice.
(C) Electron micrograph showing milk-engulfing macropinosomes (arrow) at the apical membrane in involution day 2 glands. These were not detected in secretory MECs at pregnancy day 18. Bar: 500nm.
(D) Macropinocytic engulfment of FITC conjugated casein in MEC cultures was drastically impaired upon inhibition of Rac1. Cells were treated with DMSO vehicle only (control), or 50mM NSC23766 Rac1 inhibitor overnight, prior to measurement of macropinocytosis. 70k Mr TRITC–dextran was used as a positive marker of macropinocytic engulfment. Bar: 10mm.
(E) Quantification of (A), casein and dextran positive macropinosomes were counted in 100 cells for each condition. Error bars; +/-SEM of n=3.
(F) Gene expression data from WT and *Rac1-/-* Involution day 2 glands showing down-regulation of engulfment receptor gene sets in *transgenics*, n=3 mice were used per condition.

Gene symbol	Gene description	FC	P. Value
Csn1s2a	casein alpha s2-like A	-38.63 to -45.05	1.90E-05 to 1.43E-03
Wap	whey acidic protein	-10.25	2.55E-05
Csn2	casein beta	-2.12	2.93E-03
Csnk1g3	casein kinase 1, gamma 3	-2.95	1.00E-02
Csn3	casein kappa	-4.05	1.41E-02
Csn1s2b	casein alpha s2-like B	-1.89	2.15E-02
Csnk1a1	casein kinase 1, alpha 1	-2.32	3.02E-02
Csn1s1	casein alpha s1	-4.02	4.93E-02
Btn1a1	butyrophilin, subfamily 1, member A1	-5.21	2.89E-03
Xdh	xanthine dehydrogenase	-3.86	2.03E-03
Lalba	lactalbumin alpha	-5.35	4.52E-03

Table S1 related to Fig 2: *List of lactation genes down-regulated in Rac1-/- glands.*
Microarray gene expression data from WT and *Rac1-/-* mammary glands, showing list of milk components down-regulated in *transgenics*, n=3 mice were used per condition.

Gene symbol	Gene description	FC	P. Value
Elf5	E74-like factor 5	-3.14	5.65E-03
Stat5a	signal transducer and activator of transcription 5A	-3.65	8.54E-03
Stat5b	signal transducer and activator of transcription 5B	-2.30	3.56E-02
Gata 3	GATA binding protein 3	-1.80	1.16E-01
Prlr	prolactin receptor	-1.63	3.53E-02

Table S2 related to Fig 2: *List of lactation signaling pathway genes down-regulated in Rac1-/- glands.* Microarray gene expression data from WT and *Rac1-/-* mammary glands, showing list of lactation signaling pathway components and transcription factors down-regulated in *transgenics*, n=3 mice were used per condition.

Gene	Fold Change in *Rac1-/-*
CCL7	2.61
CCL2	2.83
CCL3	2.04
CCL19	2.12
CCL22	1.44
CXCL16	1.27
CCL27	1.17
CCL4	1.38
CXCL2	1.92
CCL20	1.30
CCL8	1.57
CXCL10	1.33
CXCL13	1.10
CXCL1	1.08
CCL24	1.26
CCL17	1.30
CCL25	1.27
CCL11	1.17
CXCL11	1.16
CXCL1	1.68

Table S3 related to Fig 7: *List of chemokines up-regulated in Rac1-/- glands at involution day 2.*
(A) Microarray gene expression data from WT and *Rac1-/-* Involution day 2 mammary glands, n=3 mice were used per condition.

Supplemental Experimental Procedures

ANTIBODY	SOURCE	IF	IHC	IB
Adipophilin	Progen #GP40	1;200		
beta1 integrin	Millipore #MAB 1997	1;100		
beta-casein	in house		1;1000	1;5000
beta-casein-RRX conjugate	in house	1;200		
beta-catenin	BD Biosciences #610154	1;200		
Calnexin	Bioquote SPA-#SPC-108A/B			1;4000
Cleaved caspase-3	Cell Signalling Technology #9661	1;100		1;50
Cytokeratin 8/18	Progen #GP11	1;200		
E-cadherin (clone ECCD-2)	Life Technologies	1;100		
E-cadherin (clone 4A2)	Cell Signalling Technology mAb #14472			1;5000
Focal adhesion kinase	BD Biosciences #610088	1;100		
FAK (Y397)	Biosource Int. #44624-G	1;100		
Fibronectin	Millipore #AB2033	1;100		
F4:80 macrophage	Serotec #MCA497GA	1;200		
Green fluorescent protein	Invitrogen #A11122	1;200		1;2000
Laminin 1	in house	1;500		
Paxillin	BD Biosciences #610233	1;200		
Paxillin (Y31)	Life Technologies #44720-G	1;200		
Rac1 (clone 23A8)	Merck Millipore #05-389			1;1000
Smooth muscle actin (clone 1A4)	Sigma #A2547	1;500		
Stat5a	Santa Cruz #1081	1;200		1;5000
Stat5a (Y694)	Cell Signalling Technology #9359			1;1000
Vinculin	Sigma #V4505	1;100		
ZO1 (clone R40.76)	Millipore #MAB1520	1;200		
